# Essential Tremor and Digital Biomarkers: A Scoping Review Using the TRACE Framework to Map Readiness for Clinical Trials and Routine Practice

**DOI:** 10.5334/tohm.1205

**Published:** 2026-06-08

**Authors:** David Ledingham, Antonella Macerollo, Nicola Pavese

**Affiliations:** 1 Clinical Ageing Research Unit, Newcastle University, Campus for Ageing and Vitality, Newcastle upon Tyne, UK; 2 Neurosciences, Newcastle Upon Tyne NHS Foundation Trust, Newcastle Upon Tyne, UK; 3 Translational and Clinical Research Institute, The Medical School, Newcastle University, Newcastle Upon Tyne, UK; 4 NIHR Newcastle Biomedical Research Centre (BRC), Newcastle upon Tyne, UK; 5 The Walton Centre NHS Foundation Trust for Neurology and Neurosurgery, Liverpool, UK; 6 Institute of Systems, Molecular and Integrative Biology, The University of Liverpool, UK; 7 Department of Nuclear Medicine, Aarhus University Hospital, Aarhus, Denmark

**Keywords:** Essential tremor, Digital biomarkers, Wearable sensors, Clinical trials, TRACE framework, Scoping Review

## Abstract

**Background:**

Essential Tremor (ET) is the most prevalent movement disorder in adults yet remains underrepresented in digital biomarker research. As therapies evolve, the absence of validated digital endpoints represents a growing translational gap. We introduce TRACE (Technology Readiness And Clinical Evidence), a novel five-tier validation maturity framework, and apply it in a scoping review of the ET digital biomarker literature.

**Methods:**

A PRISMA-ScR scoping review was conducted across four databases (2000–2025). Studies were eligible if they reported quantitative digital tremor measurement in ten or more ET participants. Each study was assigned to the highest satisfied TRACE tier: technical verification (T1), referenced clinical validity (T2), ambulatory and longitudinal utility (T3), clinical trial readiness (T4), or economic and implementation readiness (T5).

**Results:**

One hundred and sixty-five studies were included: wearable inertial measurement units (n = 114), digitised handwriting (n = 16), computer vision (n = 15), surface EMG (n = 10), voice (n = 6), and gait (n = 4). One hundred and fifty-four (93%) were T2; nine T3; two T4, both deploying the Cala Health TAPS wristband; none T5. Home or ambulatory deployment was present in 13 studies, minimum detectable change data in three, and patient-reported outcome correlation in 11.

**Discussion:**

Only 11 studies across two modalities were classified beyond T2, demonstrating ambulatory or longitudinal evidence beyond supervised clinical validation; the TAPS wristband is the sole platform to achieve Tier 4. Home deployment, minimum detectable change derivation anchored to patient experience, and patient-reported outcome integration remain key prerequisites before digital tremor metrics can function as trial endpoints or inform routine practice.

**Highlights:**

Despite rapid advances in digital outcome measures for Parkinson’s disease, essential tremor remains largely overlooked. Reviewing 165 studies across six sensing modalities, we introduce TRACE, a five-tier validation framework, and find that while 93% of devices demonstrate basic clinical validity, only two achieve clinical trial readiness.

## Introduction

Essential Tremor (ET) is the most prevalent movement disorder in adults [1,2,3], yet it remains substantially underrepresented in biomarker and digital health research relative to Parkinson’s disease (PD) [4,5]. Characterised by kinetic and postural tremor [3], ET presents specific challenges for longitudinal monitoring: symptoms fluctuate with fatigue, emotional state, caffeine, and medication timing [6]; traditional clinical rating scales are episodic, subjective, and insensitive to the intra-individual variability that is central to the patient experience [7,8]. In addition, many digital biomarker studies to date have enrolled ET patients primarily as a comparator group for PD rather than as the primary population of interest [9,10,11,12,13,14,15,16,17,18,19]. As novel pharmacological agents and neuromodulatory interventions enter clinical development for ET [20,21], objective sensor-derived tremor metrics offer a promising alternative to subjective clinical scales, but their validation for use as trial endpoints or in routine practice remains incomplete [22,23].

Wearable inertial sensors, digitised drawing tasks, computer vision, surface EMG, and acoustic voice analysis have each been applied to tremor quantification in ET. However, technical feasibility in a supervised clinic setting does not automatically confer utility as a clinical trial endpoint: a metric must also demonstrate ambulatory validity, temporal stability across days, sensitivity to clinically meaningful change, and interpretability in terms of patient experience before it can serve as a primary or secondary outcome measure in a longitudinal interventional study [23,24].

Given the methodological heterogeneity of the field and our aim to map evidence breadth across multiple sensing modalities, rather than synthesizing effect sizes, we conducted a scoping review [25]. This scoping review has three objectives: to map the landscape of digital biomarkers in ET by sensor modality and context of use; to evaluate their clinical trial readiness using the TRACE framework, a novel five-tier validation maturity model developed for this review; and to identify the critical evidence gaps and propose a roadmap for advancing the most promising modalities toward deployment as endpoints in future ET trials and routine clinical practice.

## Methods

### Study Design and Registration

This scoping review was conducted in accordance with PRISMA-ScR [25]. A study protocol was developed a priori by consensus among all authors, defining eligibility criteria, search strategy, data charting fields, and tier assignment rules prior to screening. The protocol was not prospectively registered. No ethical approval was required.

### Eligibility Criteria

Studies were eligible if they met all the following: publication from 1 January 2000 onwards; original peer-reviewed research reporting quantitative digital measurement of tremor in human participants; inclusion of ET patients as a primary population or identifiable subgroup of ten or more participants; use of a digital sensor-based technology; and availability in English. Studies in which ET patients served as a comparator group remained eligible provided the ET subgroup comprised ten or more participants with extractable ET-specific data; such studies were flagged accordingly. Digital biomarkers were operationally defined as quantitative tremor metrics derived from body-worn inertial sensors, accelerometers, gyroscopes, goniometers, digitising tablets, surface electromyography, computer vision or smartphone camera systems, or acoustic recording devices, deployed for the purpose of tremor assessment or monitoring. Studies using these technologies solely for neurophysiological characterisation of tremor mechanisms without reporting extractable clinical measurement endpoints were excluded.

Studies were excluded if they were reviews, editorials, book chapters, conference abstracts without full data, or case reports; if they focused exclusively on non-digital biomarkers; or if published prior to 2000. Studies evaluating devices solely for tremor suppression were excluded unless extractable tremor signal metrics were reported before or after intervention, in which case they were included and flagged as therapeutic context studies.

An a priori exception to the ten-participant threshold was specified for repeated-measures or interventional designs in which the number of independent observations substantially exceeded the number of participants, provided the design was pre-specified. Full eligibility criteria and decision logic for borderline cases are provided in Supplementary File 1.

### Information Sources and Search Strategy

Searches were conducted across PubMed, Web of Science, Scopus, and IEEE Xplore; the last was included to capture the engineering and signal processing literature systematically underrepresented in biomedical databases. Searches were limited to 1 January 2000 to August 2025; the final search was executed on 8 September 2025. Search terms combined Essential Tremor and digital assessment technology domains using Boolean operators, deliberately excluding clinical validation or biomarker performance terms to avoid overly restrictive filtering. Reference lists of all included studies and relevant prior reviews were hand-searched. In response to peer review, a supplementary search was conducted on 27 April 2026 across the same four databases using expanded sensor-specific terminology (accelerometry, electromyography, goniometry, and related MeSH terms) to capture studies indexed under device-specific rather than digital biomarker headings. The supplementary search was restricted to the same date window (1 January 2000 to August 2025) as the original search. Full search strings are provided in Supplementary File 2.

### Study Selection

**Figure 1:**
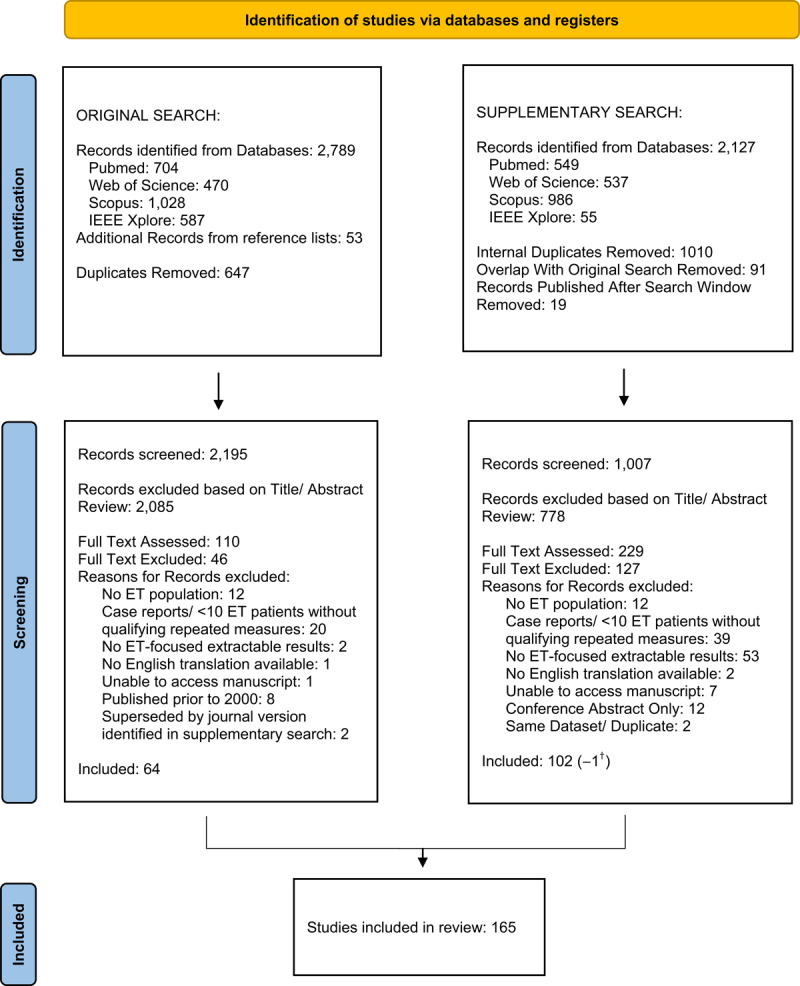
PRISMA-ScR flow diagram for the scoping review of digital biomarkers in Essential Tremor. Records were identified through systematic searching of four electronic databases and supplemented by manual reference list searching of included studies and relevant reviews. Deduplication was performed prior to title and abstract screening. Full-text articles were assessed against pre-specified eligibility criteria; reasons for exclusion are reported. The total number of unique studies included in the scoping review is 165. PRISMA-ScR, Preferred Reporting Items for Systematic Reviews and Meta-Analyses extension for Scoping Reviews; ET, Essential Tremor; IEEE, Institute of Electrical and Electronics Engineers.

Screening was conducted in two sequential stages. DL screened all titles and abstracts, retaining uncertain records for full-text review. DL then performed full-text assessment with reasons for exclusion documented. Borderline decisions were reviewed by AM or NP, with final determination by discussion. Study selection is summarised in the PRISMA-ScR flow diagram (Figure 1).

### Data Charting

A structured 27-field data charting instrument was developed a priori, covering study identity, participant characteristics, technology characterisation, context of use, outcome metrics, and TRACE tier evidence variables. It is provided in full as Supplementary File 3. Unreported fields were recorded explicitly; absence of reporting is itself informative in a field where many validation gaps are gaps of omission rather than negative findings. Data charting was performed by DL across all 165 studies. AM and NP independently reviewed all studies assigned Tier 3 or above, and all Tier 2 studies flagged as Tier 2+; disagreements were resolved by discussion. Consistent with scoping review methodology, formal critical appraisal was not performed; TRACE tier assignment captures validation maturity, not methodological rigour.

### The TRACE Framework

To evaluate the clinical trial readiness of identified digital biomarkers, we developed the TRACE (Technology Readiness And Clinical Evidence) framework, a five-tier validation maturity model in which each tier corresponds to a discrete and progressively more demanding domain of evidence. Existing frameworks, most notably the Digital Medicine Society V3 model, provide a strong foundation for sensor verification and analytical validation but group all clinical evidence into a single stage [23]. A key translational bottleneck, the’ Ecological Gap’, exists between technologies that demonstrate correlation with clinical rating scales in a controlled setting and those with proven utility in longitudinal, ambulatory, real-world contexts. TRACE addresses this by deconstructing the clinical validation phase into two distinct tiers, Referenced validity and Ambulatory utility, and extending the pathway to encompass the operational and economic evidence required for integration into clinical trials and ultimately clinical practice.

The five tiers are defined as follows. Tier 1 (Technical Verification) requires bench or phantom testing demonstrating sensor accuracy against a mechanical or electrical reference standard, independent of patient data. Tier 2 (Referenced Clinical Validity) requires demonstration of correlation with an established tremor rating scale, such as The Essential Tremor Rating Scale (TETRAS), the Fahn-Tolosa-Marin Tremor Rating Scale (FTM-RS)/the Clinical Rating Scale for Tremor (CRST) [26,27], or reliable discrimination between ET and a comparator group, in a controlled clinical or laboratory setting; both confirm that the signal captures clinically meaningful tremor variation under supervised conditions. Tier 3 (Ambulatory and Longitudinal Utility) requires evidence of sensor performance in a home or ambulatory setting across at least two separate calendar days, with demonstration of temporal stability, test-retest reliability, or sensitivity to change over time. Tier 4 (Clinical Trial Readiness) requires the digital metric to be pre-specified as a primary or secondary endpoint in a registered trial, with operational delivery demonstrated through adherence and data completeness reporting. Tier 5 (Economic and Implementation Readiness) requires formal evidence of patient acceptability alongside at least one of the following: validated health economic modelling or demonstrated integration into a reimbursed clinical workflow. Although less immediately applicable than the preceding tiers, its relevance will grow as metrics validated at Tier 4 begin transitioning from trial endpoints toward tools for guiding treatment decisions in routine practice. Each study was assigned to the highest tier for which all core criteria were satisfied. TRACE was deliberately calibrated to the current maturity of the ET field; formal FDA or EMA qualification, which represents post-hoc endorsement of an established evidence base rather than a prerequisite for trial deployment, constitutes the strongest possible evidence at Tier 4 but is not required for assignment. Studies meeting Tier 2 criteria through multi-visit or test-retest clinic designs with calendar-separated sessions but without home deployment were flagged as Tier 2+; Tier 3 studies additionally demonstrating operational features of Tier 4 readiness, such as multi-site data collection or quantified adherence, without a pre-registered digital endpoint were flagged as Tier 4 signal. Full tier definitions, core criteria, the Tier 2/3 boundary decision flowchart, and decision logic for borderline cases are provided in Supplementary File 1.

### Synthesis

Results are presented as a narrative evidence map by sensor modality and TRACE tier. No meta-analysis was performed given the heterogeneity of technologies, signal processing approaches, outcome metrics, and study designs. Quantitative summaries of study characteristics are presented alongside modality-specific narrative subsections and a structured endpoint readiness synthesis drawing on TRACE tier assignments and binary validation flags.

## Results

### Study Selection

The original database search identified 2,789 records with a further 53 from citation hand-searching. After deduplication and screening, 64 studies were included. The supplementary search retrieved 2,127 records; after deduplication and removal of overlap with the original search, 1,007 new unique records were screened, of which 102 met eligibility criteria. After reconciliation of one overlapping dataset, the combined total was 165 studies. The complete screening pathway and exclusion reasons are reported in Figure 1.

### Characteristics of Included Studies

The 165 included studies span 2000 to 2025, with publication volume accelerating markedly from 2018 onwards (62 studies before 2018; 103 from 2018 onwards), reflecting broader expansion of wearable and mobile sensing technology. Studies originated from 35 countries; the United States contributed the largest share (n = 49), followed by Germany (n = 15), Spain (n = 14), China (n = 11), and the Czech Republic (n = 7), with the remainder distributed across Europe, Asia, Australasia, the Middle East, and the Americas.

By sensing modality, wearable inertial measurement units (IMUs) and smartwatches account for 114 studies (69%), digitised handwriting and drawing for 16 (10%), computer vision for 15 (9%), surface EMG for 10 (6%), acoustic voice analysis for six (4%), and gait wearables for four (2%). Twenty-four studies deployed sensors spanning two modality categories and are listed under their primary modality. Across all modalities, most tasks and sensor placements target postural or kinetic upper-limb tremor, the cardinal manifestation of ET. Voice tremor (n = 6), gait disturbance (n = 4), and head tremor are sparsely represented, leaving a substantial portion of the ET clinical spectrum without comparable digital characterisation. Study design was predominantly single-session (122 studies); RCTs accounted for 15, multi-visit clinic designs for 14, longitudinal designs for 11, and test-retest designs for three. Studies enrolling ET as a comparator group or within mixed cohorts, and studies conducted in a therapeutic context, are flagged in Table 1.

**Table 1:** Characteristics of included studies (n = 165).


AUTHOR, YEAR	STUDY DESIGN	ET n	DEVICE/PLATFORM	CONTEXT	CLINICAL ANCHOR	TRACE TIER	NOTES/FLAGS

**Wearable IMUs (n = 114)**							

Bilodeau, 2000^†^ [50]	Longitudinal	13	Miniature accelerometer, force transducer, and surface EMG	Lab/clinic	BFRS	Tier 2	Therapeutic context

Louis, 2000^†^ [51]	Single-session	19	Modified Klove-Matthews Motor Steadiness Battery (Groove-Type Steadiness Tester and Nine-Hole Steadiness Tester by Lafayette Instrument, with impulse counters) and Quantitative Computerized Tremor Analysis Platform using triaxial accelerometers and surface EMG	Hybrid	wTRS	Tier 2	

Ondo, 2000 [52]	RCT	25	Unnamed triaxial accelerometer	Lab/clinic	BFRS	Tier 2	Therapeutic context

Wharrad, 2000 [53]	Single-session	20	Bruel and Kjaer type 4367 piezo-electric accelerometer	Lab/clinic	None	Tier 2	

O’Suilleabhain, 2001 [54]	Single-session	28	3Space Fastrak (Polhemus, Inc. Colchester, VT)	Lab/clinic	Clinician visual estimate	Tier 2	Contains Tier 1 component

Obwegeser, 2001 [55]	Multi-visit	31	MM-1 Movement Monitor (Axon Instruments, Foster City, CA)	Lab/clinic	FTM-TRS	Tier 2	ET subgroup. Therapeutic context

Brennan, 2002 [56]	Single-session	31	Ultra-light piezoresistive miniature accelerometers (±25 g, 0.5 g)	Lab/clinic	wTRS	Tier 2	

Gironell, 2002 [57]	RCT	10	Single-plane accelerometer transducer (Grass Instruments Division, Astro-Med Inc.)	Lab/clinic	TCRS	Tier 2	Therapeutic context

Zeuner, 2003^†^ [58]	Single-session	11	Four-gram triaxial piezo-resistive accelerometers (Kistler Instrument Corp.) and tin surface EMG electrodes	Lab/clinic	None	Tier 2	ET comparator

Caligiuri, 2004 [59]	Test-retest	35	Tremorometer (FlexAble Systems, Inc.) comprising a pair of dual-axis accelerometers	Lab/clinic	UPDRS	Tier 2	Contains Tier 1 component

Gironell, 2004^†^ [60]	Single-session	223	Piezoresistive single-plane Grass SPA accelerometer transducer and silver-silver chloride surface EMG electrodes	Lab/clinic	None	Tier 2	

Ushe, 2004 [61]	Single-session	16	T1 Tremor Analysis System (Neurokinetics, Edmonton, Alberta, Canada)	Lab/clinic	None	Tier 2	Therapeutic context

Bushara, 2005 [62]	RCT	24	FlexAble-Systems triaxial accelerometer	Lab/clinic	BFRS	Tier 2	Therapeutic context

Frima, 2006 [63]	RCT	10	ENTRAN Egax monoaxial accelerometer with Cardiff tremor acquisition and system analysis	Lab/clinic	None	Tier 2	Therapeutic context

Gironell, 2006 [64]	RCT	16	Tri-axial accelerometer transducer (Biopac Systems, Inc.)	Lab/clinic	TCRS	Tier 2	Therapeutic context

Herzog, 2007^†^ [65]	Single-session	10	Piezoelectric accelerometer, CED 1401 system for EMG, and 3SPACE FASTRAK magnetic six-degree-of-freedom measurement system (Polhemus Inc)	Lab/clinic	FTM-TRS	Tier 2	Therapeutic context

Zesiewicz, 2007 [66]	RCT	20	Catsys System (portable PC-based test system with an accelerometry device, Danish Product Development, Ltd)	Lab/clinic	FTM-TRS	Tier 2	Therapeutic context

Zesiewicz, 2007 [67]	RCT	22	Catsys System (portable PC-based test system with an accelerometry device, Danish Product Development, Ltd)	Lab/clinic	FTM-TRS	Tier 2	Therapeutic context

Shaikh, 2008 [9]	Single-session	35	Unnamed three-axis accelerometer	Lab/clinic	None	Tier 2	ET comparator

Matsumoto, 2009 [68]	Single-session.	17	Unnamed custom system comprising an HP Compaq tc4400 tablet PC and JCM AW-100(RX) 3-axis accelerometers	Lab/clinic	Clinician visual estimate	Tier 2	ET comparator

Costa, 2010 [69]	Single-session	18	Uniaxial accelerometer (ADXL105, Aircraft Medical/Morpheus Medical)	Lab/clinic	FTM-TRS; WEMOVE tremor scale	Tier 2	ET subgroup

Mostile, 2010 [70]	Single-session	20	Kinesia (CleveMed)	Lab/clinic	TETRAS	Tier 2	

Heldman, 2011 [71]	Single-session	10	Kinesia (CleveMed)	Lab/clinic	wTRS	Tier 2	

Muthuraman, 2011 [72]	Single-session	41	Unnamed accelerometer	Lab/clinic	None	Tier 2	ET comparator

Šprdlík, 2011 [73]	Single-session	30	MTx (Xsens)	Lab/clinic	FTM-TRS	Tier 2	

Uchida, 2011 [74]	Single-session	11	Unnamed biaxial micro-accelerometer (recorded via NEC Sanei Charge Amplifiers and C-LOGGER software)	Lab/clinic	Clinician visual estimate	Tier 2	ET subgroup

de Haas, 2012^†^ [75]	RCT	9	Three miniature linear piezo-electric accelerometers (Nihon Kohden, MT-3T) combined with Grass 15LT surface EMG	Lab/clinic	Performance-based tremor evaluation	Tier 2	Therapeutic context

Hossen, 2014^†^ [10]	Single-session	41	Unnamed system comprising a 2-gram piezoelectric accelerometer and bipolar surface-EMG with silver-silver-chloride electrodes	Lab/clinic	None	Tier 2	ET comparator

Wastensson, 2013 [76]	Single-session	22	CATSYS Tremor Pen and eurythmokinesimeter (EKM)	Lab/clinic	FTM-TRS	Tier 2	Therapeutic context

Bhidayasiri, 2014 [77]	Single-session	10	Unnamed low-cost 3-dimension inertial sensor prototype	Lab/clinic	FTM-TRS	Tier 2	ET subgroup

Gironell, 2014 [78]	RCT	10	Unnamed accelerometry system	Lab/clinic	TCRS	Tier 2	Therapeutic context

Pathak, 2014 [79]	Single-session	15	Active Cancellation of Tremor (ACT) device prototype (Lift Labs)	Lab/clinic	FTM-TRS	Tier 2	Therapeutic context

Pulliam, 2014 [28]	Test-retest	20	Kinesia HomeView (Great Lakes NeuroTechnologies)	Home	None	Tier 3*	Tier 4 signal

Ruonala, 2014^†^ [80]	Single-session	17	ME6000 biosignal monitor with Medicotest M-00-S Ag/AgCl surface electrodes and MEAC-X triaxial accelerometers	Lab/clinic	None	Tier 2	ET subgroup

Wile, 2014 [12]	Single-session	14	WIMM One smartwatch (and ENTRAN EGAS analog accelerometer for Tier 1 comparison)	Lab/clinic	Clinician visual estimate	Tier 2	ET comparator; Contains Tier 1 component

Woods, 2014 [11]	Single-session	18	HTC Desire model 8181 smartphone (Bosch BMA150 Triaxial digital acceleration sensor)	Lab/clinic	None	Tier 2	ET comparator

Heo, 2015 [81]	Single-session	18	3-D gyrosensor (L3G4200D, STMicroelectronics)	Lab/clinic	None	Tier 2	

Thanawattano, 2015 [13]	Single-session	22	6-DOF inertial measurement unit (IMU) with an in-house transmission unit and software	Lab/clinic	None	Tier 2	ET comparator

Atashzar, 2016 [82]	Single-session	13	Biometrics Ltd. kinematic measurement system	Lab/clinic	None	Tier 2	

Chockalingam, 2016 [83]	Single-session	13	Lift Pulse (smartphone application on iOS and Android devices). The study utilized a “smart hat” (a modified baseball cap with a smartphone case) to measure head tremor via the smartphone’s accelerometers	Lab/clinic	FTM-TRS	Tier 2	

Ghassemi, 2016^†^ [14]	Single-session	11	Schwarzer Topas EMG system (Natus, USA) integrating two calibrated accelerometers and bipolar Ag/AgCl surface EMG electrodes	Lab/clinic	None	Tier 2	ET comparator

Barrantes, 2017 [84]	Single-session	16	iPhone 5S (using SensorLog application)	Lab/clinic	None	Tier 2	

Chakraborty, 2017 [85]	Single-session	34	Xsens MTx integrated inertial measurement units	Lab/clinic	None	Tier 2	

di Biase, 2017 [15]	Single-session	33	APDM Opal triaxial accelerometer (test cohort, Rome); Brainvision acceleration sensor (test cohort, Cologne); multiple devices in validation cohort (previously published datasets)	Lab/clinic	None	Tier 2	ET comparator

Samotus, 2017 [82]	Longitudinal	10	Biometrics Ltd. goniometers (SG150) and torsiometer (Q150) with a Noraxon TeleMyo 2400T transmitter	Lab/clinic	FTM-TRS	Tier 2	Therapeutic context

Vittal, 2017 [29]	RCT	40	Kinesia system (Great Lakes NeuroTechnologies)	Home	wTRS; CGI-s and CGI-c.	Tier 3*	Tier 4 signal; Therapeutic context

Zheng, 2017 [30]	Test-retest	8	Pebble smartwatch and Android smartphone	Hybrid	FTM-TRS	Tier 3 *	Feasibility-grade Tier 3 (n < 15); Repeated-measures exception

Bove, 2018 [86]	Single-session	20	Triaxial accelerometers (SOMNOwatch; SOMNOmedics, Randersacker, Germany)	Lab/clinic	None	Tier 2	ET subgroup

De Jesus, 2018 [87]	Single-session	11	Kinesia (Great Lakes NeuroTechnology) and GaitRite walkway system	Lab/clinic	FTM-TRS	Tier 2	Therapeutic context

Jombík, 2018 [88]	Single-session	133	Unnamed custom-made miniature tri-axial accelerometer (two ADXL320 dual-axis analog devices embedded in synthetic resin)	Lab/clinic	None	Tier 2	ET comparator

López-Blanco, 2018 [38]	Multi-visit	34	NetMD system (Sony Smartwatch 3 paired with an ASUS Android smartphone via an investigational Android Wear OS application)	Lab/clinic	FTM-TRS	Tier 2	Tier 2+

Molparia, 2018 [31]	Longitudinal	24	Pebble Smart-watch paired with LG G2 smartphone running the Fox Insight mobile application/Intel Pharma Analytics Platform	Home	None	Tier 3*	ET comparator

Berbakov, 2019 [89]	Single-session	78	Node+ sensor platform (paired with TremorSense Android application)	Lab/clinic	None	Tier 2	ET subgroup

Kroneberg, 2019 [90]	Single-session	12	Mobility Lab (APDM)	Lab/clinic	FTM-TRS	Tier 2	ET subgroup

Lora-Millán, 2019 [91]	Single-session	18	TechMCS inertial sensors (Technaid, SP)	Lab/clinic	FTM-TRS	Tier 2	Therapeutic context

Pan, 2019 [92]	Single-session	20	Smartwatch (specific brand unnamed)	Lab/clinic	FTM-TRS	Tier 2	

Paschen, 2019 [93]	longitudinal	20	Viking, Nicolet EDX System (Natus Medical Inc/Fa. Jäger–Tönnies GmbH/VIASYS Healthcare Inc)	Lab/clinic	FTM-TRS	Tier 2	Therapeutic context

Zheng, 2019 [94]	Single-session	20	Portable human movement monitoring system (smartwatch with Android smartphone) utilizing an IoTA blockchain ledger	Lab/clinic	FTM-TRS	Tier 2	

Bruno, 2020 [95]	Single-session	10	Kinesia motion sensor (Great Lakes NeuroTechnologies Inc.)	Lab/clinic	TETRAS; FTM-TRS	Tier 2	Therapeutic context

Casamento-Moran, 2020 [96]	Single-session	29	Trigno Wireless Sensors (Delsys) and custom low-friction potentiometers (Mouser Electronics)	Lab/clinic	FTM-TRS; TETRAS	Tier 2	Therapeutic context

Loaiza Duque, 2020 [97]	Single-session	20	Built-in triaxial gyroscope of an iPhone 5S using the SensorLog application	Lab/clinic	None	Tier 2	ET subgroup

Jog, 2020 [98]	RCT	30	TremorTek investigational device	Lab/clinic	FTM-TRS	Tier 2	Therapeutic context

Isaacson, 2020 [21]	RCT	263	Cala Health wrist-worn TAPS neuromodulation device	Home	TETRAS	Tier 4**	Therapeutic context

Jombík, 2020 [88]	Single-session	75	Custom-made tri-axial linear accelerometer (made from two ADXL320 dual-axis analog devices)	Lab/clinic	FTM-TRS	Tier 2	

Kim, 2020 [99]	Single-session	9	Custom wireless wearable tremor modulation system (wrist device with LSM303D accelerometer and constant voltage stimulator)	Lab/clinic	TETRAS	Tier 2	

Kwon, 2020 [100]	Single-session	18	Unnamed wearable gyro-sensor based measurement system	Lab/clinic	FTM-TRS	Tier 2	

McGurrin, 2020 [101]	Single-session	13	APDM Opal inertial sensors	Lab/clinic	TETRAS	Tier 2	Contains Tier 1 component

Robertson, 2020 [102]	Single-session	18	ETSense™ inertial sensor tremorography device along with Kinesia Home View™	Lab/clinic	FXTAS-RS	Tier 2	ET comparator

van Brummelen, 2020 [103]	Single-session	10	Seven Consumer Product Accelerometers (Apple iPhone 7, Apple iPod Touch 5, Apple Watch 2, Huawei Nexus 6P, Huawei Watch, mbientlab MetaWear watch, mbientlab MetaWear clip) compared against a reference Laboratory-Grade Accelerometer (Biometrics ACL300)	Lab/clinic	None	Tier 2	ET subgroup

Wilkes, 2020 [104]	Single-session	20	Delsys Trigno Wireless System	Lab/clinic	TETRAS; FTM-TRS	Tier 2	Therapeutic context

Yu, 2020 [105]	Single-session	15	Tri-axial accelerometer (APDM Wearable Technologies, Portland, OR)	Lab/clinic	FTM-TRS	Tier 2	Therapeutic context

Zajki-Zechmeister, 2020 [106]	Single-session	16	TREMITAS-System (TREM) pen-shaped sensor	Lab/clinic	TETRAS	Tier 2	ET subgroup

Fuchs, 2021 [107]	Single-session	20	iPhone 5s running the TREMOR12 application	Lab/clinic	FTM-TRS	Tier 2	

Kwon, 2021 [39]	Single-session	18	Triaxial gyro sensors (L3G4200D, STMicroelectronics)	Lab/clinic	FTM-TRS	Tier 2	

Papapetropoulos, 2021 [108]	RCT	95	Kinesia ONE	Lab/clinic	TETRAS	Tier 2	Therapeutic context

Pokhabov, 2021 [109]	Single-session	30	Kolibri wireless electrophysiological signal monitoring system	Lab/clinic	None	Tier 2	ET subgroup

Brillman, 2022 [32]	Longitudinal	216	Cala Trio (Cala Health)	Home	None	Tier 3*	Tier 4 signal; Therapeutic context

Everlo, 2022† [110]	Single-session	90	Brain RT software utilizing accelerometers and surface electromyography (polymyography)	Lab/clinic	Clinician visual estimate	Tier 2	ET subgroup

Gauthier-Lafreniere, 2022 [111]	Single-session	25	GENEActiv Original wristwatch (Activinsights, UK) and a custom MATLAB application	Lab/clinic	CRST	Tier 2	

Kovalenko, 2022^†^ [112]	Single-session	13	SensorTile wearable platform (STMicroelectronics) and Logitech BRIO 4k video camera	Lab/clinic	none	Tier 2	ET comparator

Ma, 2022 [113]	Single-session	98	Unnamed miniature IMU device (utilizing a QFN packaged multi-chip MPU-9250)	Lab/clinic	CRST	Tier 2	

Ma, 2022 [114]	Single-session	54	IMU-based wearable device (JY901, BMI160)	Lab/clinic	CRST	Tier 2	

McGurrin, 2022 [33]	Longitudinal	13	Unnamed wearable inertial sensors	Hybrid	TETRAS	Tier 3*	Feasibility-grade Tier 3

Ni, 2022 [115]	Single-session	20	Unnamed smartwatch embedded with a tri-axis accelerometer	Lab/clinic	FTMTRS	Tier 2	

Purrer, 2022^†^ [116]	Multi-visit	37	Dantec Keypoint.NET utilizing uniaxial accelerometry and surface electromyography	Lab/clinic	CRST	Tier 2	Tier 2+

Sahin, 2022 [117]	Single-session	17	Apple iPhone 8 smartphone (using Medotemic’s app Medoclinic) and LPMS-B2 STD high-resolution miniature inertia sensor	Lab/clinic	FTM-TRS	Tier 2	ET subgroup

Xing, 2022^†^ [118]	Single-session	141	Dantec Keypoint G4 (Natus Medical Inc.), combining distal finger accelerometers and forearm surface electromyography	Lab/clinic	None	Tier 2	ET comparator

Ali, 2023 [119]	Single-session	17	Delsys Trigno wearable IMU	Lab/clinic	FTM-TRS	Tier 2	

Dai, 2023 [37]	RCT	276	Cala Trio (Cala Health)	Home	BFRS	Tier 4**	Therapeutic context

Li, 2023 [120]	Single-session	12	Unnamed wearable multi-sensor measurement system (utilizing MPU9250 units)	Lab/clinic	MDS-UPDRS	Tier 2	ET comparator

Lin, 2023 [17]	Single-session	80	MATRIX (GYENNO SCIENCE)	Lab/clinic	None	Tier 2	ET comparator

Loaiza Duque, 2023 [121]	Single-session	76	TremorSoft app (using built-in smartphone 6-axis inertial sensors or an Xsens DOT wearable sensor)	Lab/clinic	None	Tier 2	ET subgroup; Tier 4 signal.

Lu, 2023 [34]	Longitudinal	808	Cala Trio™ (Cala Health)	Home	None	Tier 3*	Therapeutic context; Tier 4 signal

Metzner, 2023^†^ [122]	Single-session	20	Trigno IM Sensors and Trigno EMG Sensors (Delsys Inc.)	Lab/clinic	TETRAS	Tier 2	Therapeutic context

Pascual-Valdunciel, 2023^†^ [123]	Single-session	12	Technaid S.L IMUs and Quattrocento OT Bioelettronica bio-signal amplifier	Lab/clinic	None	Tier 2	

Piarroux, 2023^†^ [124]	Single-session	18	Unnamed polymyographic system (four-way surface electromyogram coupled with accelerometry)	Lab/clinic	None	Tier 2	None

Smid, 2023 [125]	Single-session	13	MMA8452Q tri-Axis accelerometer (Freescale Semiconductor, Inc.) with LabVIEW	Lab/clinic	FTM-TRS	Tier 2	None

van der Linden, 2023 [126]	Single-session	16	BrainProducts triaxial accelerometer (FingerACC) and GENEActiv Original wristwatch accelerometer (WristACC)	Lab/clinic	MDS-UPDRS	Tier 2	ET subgroup; Therapeutic context

Vescio, 2023 [127]	Single-session	40	RT-Ring (utilizing an ST Microelectronics LSM6DSL 6-axis IMU)	Lab/clinic	None	Tier 2	ET subgroup

Ameer, 2024 [128]	Single-session	45	Unidirectional piezoelectric accelerometer analyzed via Natus KEYPOINT.NET Software v. 2.40	Lab/clinic	TETRAS	Tier 2	

Cabral, 2024 [129]	Single-session	18	Custom wearable device (incorporating inertial measurement units and piezoelectric actuators)	Lab/clinic	FTM-TRS	Tier 2	Therapeutic context

Hollý, 2024 [130]	Single-session	40	MTw Awinda triaxial accelerometers (Xsens, the Netherlands)	Lab/clinic	TETRAS	Tier 2	

Hubená, 2024 [131]	Single-session	25	MTw Awinda (Xsens, the Netherlands)	Lab/clinic	TETRAS	Tier 2	ET comparator

Tang, 2024^†^ [132]	Single-session	53	Dantec Keypoint signal acquisition system	Lab/clinic	FTM-TRS	Tier 2	ET subgroup

Zhang, 2024 [133]	Single-session	20	Unnamed smartwatch monitoring system	Lab/clinic	FTM-TRS	Tier 2	

Aladro, 2025 [35]	Longitudinal	12	Unnamed portable human movement monitoring system (comprising a smartwatch with a tri-axis accelerometer and a paired Android smartphone app)	Home	FTM-TRS	Tier 3*	Feasibility-grade Tier 3; Therapeutic context

Bártová, 2025^†^ [134]	Multi-visit	12	Unnamed accelerometer and camera setup (Note: A URIS I neuromodulation system was used for the therapeutic intervention)	Lab/clinic	TETRAS	Tier 2	Therapeutic context

Buonocore, 2025 [18]	Single-session	21	RT-ring	Lab/clinic	None	Tier 2	ET comparator

Häring, 2025 [135]	Single-session	188	Unnamed triaxial and monoaxial accelerometers	Lab/clinic	None	Tier 2	

Mugge, 2025 [136]	Single-session	24	MTw Awinda (Xsens Technologies B.V.)	Lab/clinic	TETRAS	Tier 2	Therapeutic context

Purrer, 2025 [137]	Multi-visit	35	SOMNOwatch™ plus® (SOMNOmedics, Randersacker, Germany)	Lab/clinic	CRST	Tier 2	ET subgroup. Therapeutic context

Samiee, 2025 [138]	RCT	88	Wearable wristband (Pishgaman Rah Salamat Pars)	Lab/clinic	TETRAS	Tier 2	Therapeutic context

Tarlaci, 2025 [139]	Single-session	14	Vibration Meter app (EXA Tools, Poland) installed on a mobile phone (Vestel Venus, Android 4.0)	Lab/clinic	BFRS	Tier 2	Therapeutic context

Tsuboi, 2025 [140]	Single-session	27	Triaxial accelerometers (Logical Product, Fukuoka, Japan)	Lab/clinic	CRST	Tier 2	ET subgroup

**Digitised Handwriting (n = 16)**							

Haubenberger, 2011 [40]	Single-session	9	Wacom Intuos 3 Model PTZ-930 digitizing tablet + Neuroglyphics software	Lab/clinic	FTM-TRS	Tier 2	Contains Tier 1 component

Louis, 2012 [141]	Single-session	145	Wacom Intuos 3 digitizing tablet	Lab/clinic	Clinician visual estimate	Tier 2	

Kragelj, 2014 [142]	Single-session	15	Unnamed programmed graphical tablet with tablet pen/Computer Assisted Spirography (CAS) system	Lab/clinic	None	Tier 2	

Elble, 2017 [41]	Test-retest	18	Wacom Intuos 3 digitizing tablet	Lab/clinic	FTM-TRS	Tier 2	Tier 2+

Legrand, 2017 [143]	Multi-visit	13	Wacom, Bamboo Fun Medium Pen&Touch digitising tablet	Lab/clinic	BFRS	Tier 2	Tier 2+

Schuhmayer, 2017^†^ [144]	Single-session	40	Neuroglyphics software on a Windows-based tablet-PC, and an unnamed uniaxial accelerometer	Lab/clinic	TETRAS	Tier 2	

Tam, 2017 [145]	Single-session	12	MRI-compatible touch tablet and stylus	Lab/clinic	None	Tier 2	

Kim, 2018 [146]	Single-session	161	Wacom Intuos4 graphics tablet with a wireless inking pen	Lab/clinic	None	Tier 2	

Lin, 2018 [147]	Single-session	12	Wacom Cintiq 13HD graphic tablet with a custom-made computer program	Lab/clinic	Clinician visual estimate	Tier 2	ET subgroup

Merchant, 2018 [148]	Single-session	19	Wacom Intuos 2-4 graphics tablet with wireless inked pen	Lab/clinic	FTM-TRS	Tier 2	

Sanderson, 2020 [19]	Single-session	12	Touchscreen tablet (iPad running iOS v.11.4 with a custom Swift application) paired with a pressure-sensing stylus	Lab/clinic	None	Tier 2	ET comparator

López-Blanco, 2021 [149]	Single-session	31	BQ Aquaris E4.5 Android smartphone running an under-development Android application	Lab/clinic	FTM-TRS	Tier 2	

Motin, 2021 [150]	Single-session	19	Wacom Intuos Pro Large digital tablet with a pressure-sensor mounted ink-pen	Lab/clinic	FTM-TRS	Tier 2	

Kim, 2022^†^ [151]	Single-session	11	3D gyration mouse (Air Mouse Go Plus, Gyration Inc.) and a custom-designed wrist device (LSM303D three-axis accelerometer)	Lab/clinic	TETRAS	Tier 2	

Rajan, 2023 [43]	Single-session	25	Unnamed in-house automated analysis algorithm processing images from a commercial scanner	Lab/clinic	BFRS; FTM-TRS	Tier 2	ET subgroup

Figura, 2024 [152]	Multi-visit	11	Digitising tablet (Intuos series, Wacom) running custom-acquisition software	Lab/clinic	FTM-TRS	Tier 2	ET subgroup. Therapeutic context

**Computer Vision (n = 15)**							

Deuschl, 2000 [153]	Single-session	26	MacReflex version 3.2 passive infrared movement analysis system (Qualisys, Sweden)	Lab/clinic	FTM-TRS	Tier 2	

Uhríková, 2011^†^ [154]	Single-session	26	TremAn (a software tool for computer analysis of video sequences) and Xsens MTx inertial measurement units	Lab/clinic	FTM-TRS	Tier 2	

Geiger, 2018 [155]	Single-session	10	trakSTAR electromagnetic motion capture system (Ascension Technologies)	Lab/clinic	FTM-TRS	Tier 2	

Ishii, 2020 [156]	Single-session	24	Unnamed smartphone-based application with a server-side convolutional neural network (CNN)	Lab/clinic	TETRAS	Tier 2	ET subgroup

Seedat, 2020 [157]	Single-session	669	ResNet-32 convolutional neural network (CNN)	Lab/clinic	None	Tier 2	ET subgroup

Kovalenko, 2021 [16]	Single-session	13	Logitech BRIO 4K PRO camera with OpenPose library for keypoint extraction	Lab/clinic	None	Tier 2	ET comparator

Ismail, 2022 [158]	Multi-visit	12	Smartphone camera with a publicly available Instagram filter (“steady-hand filter”)	Lab/clinic	TETRAS	Tier 2	Tier 2+

Ma, 2023 [159]	Single-session	61	Portable video camera/smartphone camera utilizing a Transformer-based HRNet-DARK architecture (HRTNet-Dark) and a Tremor Detection Transformer (TDT) algorithm	Lab/clinic	CRST	Tier 2	

Wang, 2023 [160]	Single-session	50	Scanned paper drawings analyzed via an optimized Convolutional Neural Network (CNN).	Lab/clinic	None	Tier 2	

Baek, 2024 [36]	Longitudinal	37	Scanned conventional paper-and-pen drawings analyzed via custom IDL software	Hybrid	CRST	Tier 3*	ET subgroup; Tier 4 signal: adherence and data completeness quantified for home follow-up phase

Costa, 2024 [161]	Longitudinal	36	SMART motion system (BTS Engineering)	Lab/clinic	FTM-TRS	Tier 2	Tier 2+

Friedrich, 2024 [162]	Multi-visit	66	Mediapipe and DeepLabCut (DLC-RCNN) computer vision pose-tracking algorithms	Lab/clinic	FTM-TRS	Tier 2	Therapeutic context

Lee, 2024 [163]	Single-session	59	In-house tremor measurement app utilizing an Android-based smartphone camera (Samsung Galaxy S20)	Lab/clinic	CRST	Tier 2	Contains Tier 1 component

Shin, 2025^†^ [164]	Single-session	22	DeepLabCut (marker-less deep learning pose estimation) alongside an Xsens DOT gyroscope used for convergent validation	Lab/clinic	TETRAS; TWSTRS-2	Tier 2	ET comparator

Wolke, 2025 [165]	Single-session	13	Mediapipe (by Google) and Vision (by Apple) computer vision frameworks utilizing a smartphone camera (Apple iPhone 12 mini)	Lab/clinic	TETRAS	Tier 2	Contains Tier 1 component

**Surface EMG (n = 10)**							

Akbostanci, 2000 [166]	Single-session	100	San-ei EEG 1A96 electroencephalograph with Ag-AgCl surface electrodes	Lab/clinic	BFRS	Tier 2	None

Breit, 2008 [167]	Single-session	24	Unnamed long-term ambulatory surface EMG system	Home	None	Tier 2	

Nisticò, 2011 [168]	Single-session	15	Unnamed system comprising two pairs of surface electrodes and needle electrodes	Lab/clinic	None	Tier 2	ET subgroup. Tier 2+

Ohye, 2012 [169]	Multi-visit	13	Unnamed surface electromyography (EMG)	Lab/clinic	UPDRS	Tier 2	ET subgroup. Therapeutic context

Ruonala, 2013 [170]	Single-session	17	ME6000 biosignal monitor with Medicotest M-00-S Ag/AgCl surface electrodes	Lab/clinic	None	Tier 2	ET subgroup

Tavakkoli, 2014 [171]	Single-session	20	Power-Lab system	Lab/clinic	None	Tier 2	ET subgroup

Cernera, 2021 [172]	Single-session	10	Trigno Acquisition Unit wearable sensors (Delsys, Inc) and Nexus-D telemetry wand (Medtronic)	Lab/clinic	FTM-TRS	Tier 2	

Muruzheva, 2022 [173]	Single-session	90	Not reported (described only as a pair of surface electrodes for electromyographic recordings)	Lab/clinic	FTM-TRS	Tier 2	None

Gulati, 2024^†^ [174]	Single-session	28	Natus Elite – Viking six-channel surface electromyography system and a triaxial accelerometer	Lab/clinic	None	Tier 2	ET subgroup

Salazar, 2025^†^ [175]	Multi-visit	18	Unnamed surface electromyography (EMG) and accelerometer	Lab/clinic	TETRAS	Tier 2	Therapeutic context

**Acoustic Voice Analysis (n = 6)**							

Akkunje, 2021 [176]	Single-session	69	Computerized Speech Labs 4500 (KAYPENTAX) with high-fidelity microphone (SHURE SM-48) and electroglottography (EGG) model 6103 (KAYPENTAX)	Lab/clinic	VTRS	Tier 2	

Suppa, 2021 [42]	Multi-visit	58	High-definition audio recorder H4n Zoom with a Shure WH20 Dynamic Headset Microphone.	Lab/clinic	FTM-TRS	Tier 2	Tier 2+

Rao, 2023 [177]	Single-session	20	CSL4500 microphone with MDVP software (KAYPENTAX) and a custom Probabilistic Source-Filter Model (PSFM) algorithm	Lab/clinic	None	Tier 2	

Larner, 2024 [178]	Multi-visit	12	Voice Evaluation Suite (Estill Voice International) using a handheld Ultralink ULM300M wireless microphone (Behringer) and Praat Speech Analysis program	Lab/clinic	Clinician visual estimate	Tier 2	Therapeutic context

Borders, 2025 [179]	Multi-visit	18	The Computerized Speech Lab system (CSL Model 4500B, PENTAX Medical) with an AKG P220 microphone and Praat software	Lab/clinic	QUEST-Voice	Tier 2	ET subgroup. Therapeutic context.

Rusz, 2025 [180]	Single-session	54	Head-mounted condenser microphone (Beyerdynamic Opus 55) and MATLAB software	Lab/clinic	TETRAS	Tier 2	ET subgroup

**Gait & Whole-Body (n = 4)**							

Moon, 2020 [181]	Single-session	43	APDM Opal IMU sensors (analyzed via Mobility Lab software),	Lab/clinic	None	Tier 2	ET subgroup

Kroneberg, 2022 [182]	Single-session	20	Mobility Lab V1 hardware (APDM, Oregon USA)	Lab/clinic	SARA; FTM-TRS	Tier 2	Therapeutic context

Robertson-Dick, 2023 [183]	Single-session	20	APDM Mobility Lab™ six inertial sensor system (APDM™; version 1)	Lab/clinic	FXTAS-RS	Tier 2	ET comparator

Kroneberg, 2024 [184]	Single-session	12	Mobility Lab™ system (APDM, V1 hardware)	Lab/clinic	None	Tier 2	ET comparator


Studies are grouped by sensor modality and ordered chronologically within each group. TRACE tier assignments reflect the highest level of validation evidence achieved by each study. * Denotes Tier 3 (Ambulatory and Longitudinal Validation), defined as studies meeting all three criteria of calendar day separation, home or ambulatory data collection, and temporal performance evidence; ** Denotes Tier 4 (Clinical Trial Readiness), defined as a digital metric pre-specified as a primary or secondary endpoint in a registered clinical trial, or multi-site deployment across three or more independent sites with adherence and data completeness reporting. No study achieved Tier 5 (Economic and Implementation Readiness). ^†^ Denotes studies deploying sensors spanning two modality categories; listed under primary modality. ET n refers to the number of Essential Tremor participants only; where studies enrolled mixed diagnostic cohorts, participants with other diagnoses are excluded from this count. The Clinical Anchor column records the validated clinical rating scale used as the reference standard for digital metric validation. None indicates no formal correlation with a validated clinical scale was reported. Informal estimate indicates that a clinician visual rating was used without a formally validated instrument. Where a scale was used solely for patient characterisation or group assignment without direct correlation against the digital metric, this is also recorded as None. The Notes and Flags column records the following methodological designations. ET comparator: ET patients were recruited as a reference group for a study whose primary focus was another movement disorder, most commonly Parkinson’s disease; ET-specific digital biomarker performance was not a primary study aim. ET subgroup: ET patients were included within a larger mixed-diagnosis cohort without a dedicated ET-focused analysis. Tier 2+: a multi-visit or test-retest clinic design providing reliability, MDC, or longitudinal validation data across calendar-separated sessions without a home or ambulatory component; these studies are assigned Tier 2 in all analyses but represent the strongest candidates for Tier 3 promotion given the addition of a home deployment component. Tier 4 signal: a Tier 3 study that additionally demonstrates one or more operational features consistent with Tier 4 readiness, such as multi-site data collection or quantified adherence reporting, but does not meet the full Tier 4 threshold due to absence of a pre-registered endpoint or defined measurement context. Feasibility-grade Tier 3: all Tier 3 criteria are met but the ET sample size is fewer than 15, limiting the generalisability and statistical precision of the longitudinal and ambulatory evidence. Repeated-measures exception: the study was included despite an ET sample size below the standard eligibility threshold of ten participants based on a strong repeated-measures design providing substantial within-subject measurement. Therapeutic context: the device serves a primary therapeutic function; the digital biomarker is a secondary or co-primary endpoint rather than the sole purpose of the device. Contains Tier 1 component: the study includes both bench-level or phantom device validation and ET clinical patient data; the tier assigned reflects the highest tier achieved in the clinical component. ET, Essential Tremor; TRACE, Technology Readiness and Clinical Evidence framework; IMU, inertial measurement unit; EMG, electromyography; TETRAS, Tremor Research Group Essential Tremor Rating Assessment Scale; FTM-TRS, Fahn-Tolosa-Marín Tremor Rating Scale; CRST, Clinical Rating Scale for Tremor; CRST-C, Clinical Rating Scale for Tremor Part C; MDS-UPDRS, Movement Disorder Society Unified Parkinson’s Disease Rating Scale; MDC, Minimum Detectable Change; NR, not reported; TAPS, Transcutaneous Afferent Patterned Stimulation; wTRS, Washington Heights-Inwood Genetic Study of Essential Tremor (WHIGET) tremor rating scale; TCRS, Tremor Clinical Rating Scale, a modified Fahn-Tolosa-Marin scale; FXTAS-RS, Fragile X-associated tremor/ataxia syndrome Rating Scale; BFRS, Bain & Findley Rating Scale; VTRS, Vocal Tremor Rating Scale; SARA, Scale for the assessment and rating of ataxia; QUEST-Voice, a 5-point patient-reported severity scale from the Quality of Life in Essential Tremor Questionnaire.

**Figure 2:**
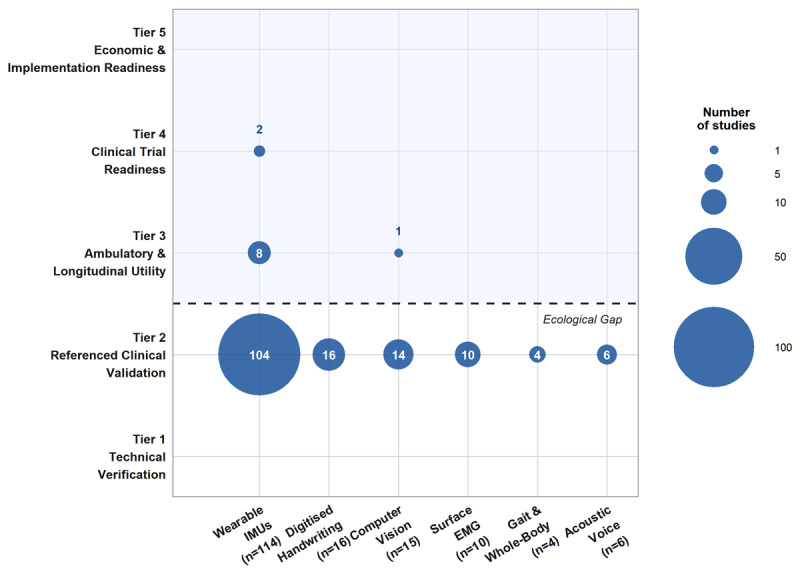
Distribution of included studies (n = 165) by sensor modality and TRACE validation tier. Each bubble represents the number of studies at a given modality and tier intersection; bubble area is proportional to the number of studies. No studies achieved Tier 5. Twenty-four studies deployed sensors spanning two modality categories and are counted once under their primary modality; had these been dual-counted, Tier 2 totals would increase by 16 for surface EMG, 6 for wearable IMUs, and 2 for computer vision. IMU, inertial measurement unit; TRACE, Technology Readiness and Clinical Evidence framework; Tier 1, Technical Verification; Tier 2, Referenced Clinical Validation; Tier 3, Ambulatory and Longitudinal Utility; Tier 4, Clinical Trial Readiness; Tier 5, Economic and Implementation Readiness.

Against the TRACE framework, 154 of 165 studies (93%) are classified at Tier 2 (Table 1), demonstrating construct or discrimination validity in a supervised clinical setting. Nine reach Tier 3 [28,29,30,31,32,33,34,35,36], all IMU-based except one using remote optical analysis of posted drawing sheets [36]; two achieve Tier 4, both deploying the Cala Health TAPS wristband [21,37]. No study meets Tier 5 criteria. Within the Tier 2 evidence base, eight studies employed multi-visit or test-retest clinic designs providing reliability or longitudinal validation data across calendar-separated sessions without a home component (Tier 2+); these are highlighted in Table 1. Five Tier 3 studies additionally met operational prerequisites for Tier 4 through multi-site delivery or quantified adherence reporting but lacked a pre-registered tremor endpoint and are flagged as Tier 4 signal studies [28,32,34]. The modality-by-tier distribution is visualised in Figure 2.

### Modality Findings

#### Wearable IMUs and Smartwatches (n = 114)

**Figure 3:**
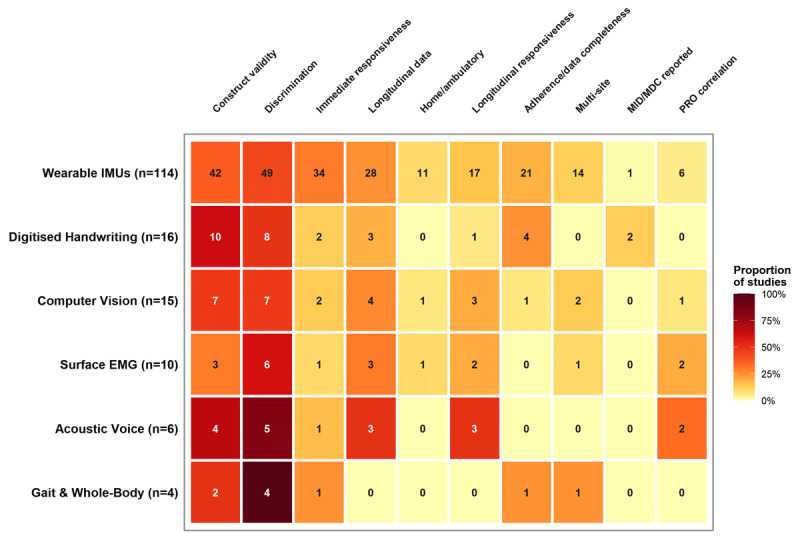
Evidence gap matrix for digital biomarker validation in Essential Tremor across six sensor modalities (n = 165). Rows represent the six sensor modalities identified in this review. Columns represent ten binary validation criteria extracted from each included study. Cell colour indicates the proportion of studies within each modality meeting that criterion, scaled from yellow (low proportion) to dark red (high proportion); cell values display the raw count. Twenty-four studies deployed sensors spanning two modality categories and are counted once under their primary modality; validation flags are reported from the primary modality only. MID, minimum important difference; MDC, minimum detectable change; PRO, patient-reported outcome; IMU, inertial measurement unit; EMG, electromyography.

The IMU evidence base is the largest and most technically heterogeneous. Construct validity against clinical rating scales is demonstrated in 42 studies; discrimination validity between diagnostic groups in 49 (Table 1; Figure 3). Study design is predominantly single-session; 14 RCTs and eight longitudinal designs provide multi-visit data, though most were conducted in a therapeutic rather than validation context (Table 1). López-Blanco and colleagues reported MDC values for a smartwatch gyroscope in a two-visit clinic design (Tier 2+) [38]; PRO correlation is present in six studies (Figure 3).

Eight studies reach Tier 3, all demonstrating ambulatory home monitoring or longitudinal data collection across calendar-separated days (Table 1; Figure 2). The Cala Health TAPS platform accounts for five of these [21,29,32,34,37]. Three carry Tier 4 signal flags [28,32,34]; two reach Tier 4 [21,37]. The two Tier 4 studies, both deploying the Cala Health TAPS wristband, represent the strongest validation evidence in the review. Isaacson and colleagues evaluated 263 patients in a registered multi-site trial, capturing pre- and post-stimulation tremor power twice daily over three months of home use; 21,806 valid sessions were recorded and tremor power tracked clinical rating scale improvement longitudinally (TETRAS r = 0.67) [21]. Dai and colleagues conducted a randomised pragmatic trial (n = 276 completing one-month follow-up) in which tremor power was pre-specified as the primary digital endpoint (NCT05540626), with multi-site recruitment, quantified adherence, and anchoring against the BF-ADL activities of daily living scale [37]. Together these studies demonstrate that a digital tremor metric can be operationalised as a pre-registered endpoint in a registered trial with adherence and data completeness reporting at scale, the defining requirements of Tier 4. Kwon and colleagues found that wrist roll and yaw outperformed finger and forearm placements across three simultaneous sites, with direct implications for sensor positioning in trial protocols [39]. Despite the size of the evidence base, home deployment is present in only 11 of 114 studies, longitudinal responsiveness in 17, and MDC data in one (Figure 3).

#### Digitised Handwriting and Drawing (n = 16)

Construct validity is demonstrated in ten studies, predominantly against FTM-TRS or TETRAS; discrimination in eight (Table 1; Figure 3). The Wacom Intuos tablet and Archimedes spiral task are the dominant platform (Table 1). Haubenberger and colleagues established foundational validation in a pre-registered ethanol challenge design [40]. Elble and Ellenbogen derived MDC values comparable to FTM-TRS from a three-day clinic trial, noting a day-one practice effect with direct implications for trial baseline measurement (Tier 2+) [41]. No study has deployed in the home setting and no minimum important difference exists; available MDC values quantify measurement precision, not patient-perceived meaningful change.

#### Computer Vision (n = 15)

Construct validity is demonstrated in seven studies; discrimination in seven (Table 1; Figure 3). One study reaches Tier 3: Baek and colleagues analysed drawings completed at home and returned by post with follow-up to 12 months post-thalamotomy (CRST Part C R² = 0.49, 35 of 39 eligible patients returning sheets), demonstrating longitudinal responsiveness, PRO correlation, and quantified adherence (Tier 4 signal) [36]. No commercially validated contactless platform currently exists.

#### Surface EMG (n = 10), Voice (n = 6), and Gait (n = 4)

These three modalities contribute 20 studies, all at Tier 2 and predominantly single-session in design (Table 1; Figure 3). sEMG studies are exclusively lab-based; construct validity is demonstrated in three, discrimination in six (Figure 3). Voice studies include the broadest single-study validation profile in the review outside IMU: Suppa and colleagues demonstrated discrimination, treatment responsiveness across separated visits, and PRO correlation via the Voice Handicap Index (Tier 2+) [42]. Gait studies demonstrate construct validity in two and discrimination in four but are limited to single-session designs (Figure 3). The digital characterisation of voice tremor, head tremor, and gait disturbance remains disproportionately sparse relative to their clinical significance in ET and ET-plus.

### Cross-Cutting Evidence Gaps

Figure 3 maps ten validation fields against all six modalities. Evidence concentrates in construct and discrimination validity (the Tier 2 criteria) while the ecological and interpretability fields are sparse. Home or ambulatory data collection is present in 13 of 165 studies, all IMU or computer vision; longitudinal responsiveness to change in 26; adherence or data completeness reporting in 27; multi-site deployment in 18; MID or MDC data in three, all quantifying measurement precision rather than patient-perceived meaningful change; and PRO correlation in 11. Stratified analysis within the Tier 2 classification reveals an important internal gradient: eight Tier 2+ studies provide test-retest reliability or MDC data across calendar-separated clinic visits without a home component; five Tier 4 signal studies, all at Tier 3, combine ambulatory deployment with multi-site data collection or quantified adherence reporting but lack a pre-registered digital tremor endpoint (Table 1).

Two further cross-cutting limitations warrant explicit statement. First, diagnostic criteria for ET are inconsistently reported, particularly in engineering publications where enrolment is frequently described only as “diagnosed by a neurologist,” without specifying consensus criteria or whether ET-plus variants were included. Second, the phenotypic scope of the literature is narrow: upper-limb postural and kinetic tremor is addressed across all modalities, while voice, head, gait, and the non-tremor features of ET-plus are digitally under-characterised. Table 2 summarises endpoint readiness by modality.

**Table 2:** Endpoint readiness summary by sensor modality for digital biomarker validation in Essential Tremor (n = 165).


MODALITY	STUDIES (n)	HIGHEST TRACE TIER	BEST-EVIDENCED DEVICE(S)/PLATFORM(S)	PRIMARY GAP FOR TRIAL INTEGRATION	MID REPORTED (STUDY)	PRO CORRELATION (STUDY)

Wearable IMUs & Smart-watches	114	Tier 4	**Cala Trio/TAPS wristband (Cala Health)** Tier 4: Isaacson 2020 [21] (n = 263, 26 sites, home, RCT), Dai 2023 [37] (n = 276, multi-site, home, RCT). **Kinesia HomeView (Great Lakes NeuroTechnologies)** Tier 3: Pulliam 2014 [28] (n = 19, multi-site, home). Multiple Tier 3 studies also used smartwatches (Zheng 2017 [30]), unnamed wearables (McGurrin 2022^†^ [33], Aladro 2025 [35]), and Cala Trio in therapeutic context (Brillman 2022 [32], Lu 2023 [34]). 22 studies used dual modalities (See Table 1).	Formal MID absent for all platforms; MDC reported only by López-Blanco 2018 [38]. PRO correlation established in six studies (Fuchs 2021 [107], Louis 2000^†^ [51], McGurrin 2022 [33], Brillman 2022 [32], Holly 2024 [130], Aladro 2025 [35]) but none pre-specified as a primary endpoint. 105 of 114 studies are Tier 2 studies.	Yes: López-Blanco 2018 [38] (MDC only, not formally derived MID)	Yes: Fuchs 2021 [107] (QUEST); Louis 2000^†^ [51] (Tremor Disability Questionnaire); McGurrin 2022 [33] (TETRAS-ADL); Brillman 2022 [32] (PGI-I); Holly 2024 [130] (TETRAS-ADL); Aladro 2025 [35] (self-rated tremor VAS)

Digitised Handwriting & Drawing	16	Tier 2	**Wacom Intuos series (digitising tablet)**: Multiple Tier 2 studies including Haubenberger 2011 [40], Elble 2017 [41], Legrand 2017 [143], Motin 2021 [150]. Two studies used dual modalities (Schuhmayer 2017^†^ [144], Kim 2022^†^ [151]).	No home deployment using a digital tablet; no longitudinal disease-tracking study; MDC available (Elble 2017 [41], Rajan 2023 [43]) but no formally derived MID; no PRO correlation. All 16 studies are Tier 2.	Yes: Elble 2017 [41] (MDC, 51% of baseline); Rajan 2023 [43] (MDC, 19.9% for Meijer severity index)	No

Computer Vision & Contactless	15	Tier 3	No commercially available or clinically validated platform. Studies use bespoke smartphone applications (Ishii 2020 [156]; Lee 2024 [163]), repurposed social media filters (Ismail 2022 [158]; Instagram “steady-hand filter”), scanned paper drawing pipelines (Baek 2024 [36], Tier 3; Wang 2023 [160]), or open-source computer vision frameworks (Wolke 2025; Google MediaPipe/Apple Vision). Two studies used dual modalities (Uhríková 2011^†^ [154], Shin 2025^†^ [164]).	No continuous passive ambulatory monitoring; small evidence base (n = 15); no MID reported; Tier 3 evidence method-specific (mailed paper drawings, Baek 2024 [36]). Tier 4 signal present (Baek 2024 [36]: adherence quantified, longitudinal responsiveness demonstrated) but no commercial platform or pre-specified endpoint. Fourteen of 15 studies are Tier 2.	No	Yes: Baek 2024 [36] (CRST-C self-report)

Surface EMG	10	Tier 2	**Delsys Trigno wearable sensors** (Cernera 2021 [172]); **Natus Elite Viking** (Gulati 2024^†^ [174]); **ME6000 biosignal monitor** (Ruonala 2013 [170]); unnamed long-term ambulatory EMG (Breit 2008 [167]). Two studies used dual modalities (Gulati 2024^†^ [174], Salazar 2025^†^ [175]).	No home or ambulatory deployment; no longitudinal disease-tracking data; no MID reported. Construct validity typically anchored against accelerometry rather than a clinical rating scale (6 of 10 studies report discrimination validity). All studies are Tier 2.	No	Yes: Akbostanci 2000 [166] (ADL inventory); Muruzheva 2022 [173] (ADL questionnaire)

Acoustic Voice Analysis	6	Tier 2	**Zoom H4n recorder with Shure WH20 headset microphone:** (Suppa 2021 [42]) **CSL 4500B (PENTAX Medical):** used in multiple studies (Akkunje 2021 [176]; Rao 2021 [177]; Borders 2025 [179]).	No home or ambulatory recording; no continuous monitoring paradigm; evidence base remains small (n = 6). Three studies provide longitudinal data (Suppa 2021 [42], Larner 2024 [178], Borders 2025 [179]). Borders 2025 [179] applies an externally derived perceptual threshold of 4 Hz to interpret acoustic change, but this does not constitute a formally derived MDC or MID. All studies are Tier 2.	No	Yes: Suppa 2021 [42] (VHI); Borders 2025 [179] (QUEST-Voice)

Gait & Whole-Body Wearables	4	Tier 2	**APDM Opal/Mobility Lab (Clario):** used in all four studies (Moon 2020 [181]; Kroneberg 2022 [182]; Robertson-Dick 2023 [183]; Kroneberg 2024 [184]).	No home or ambulatory deployment; no longitudinal data; no MID reported; no PRO correlation. ET recruited primarily as a comparator group for PD or ataxia rather than as the target population. No ET-specific tremor metric; outcomes focus on gait and balance parameters. All studies are Tier 2.	No	No


For each modality, the highest TRACE validation tier achieved by any included study is reported alongside the best-evidenced device(s) or platform(s), the primary gap preventing trial integration, and the availability of minimum important difference and patient-reported outcome correlation data. Studies marked with † used sensors spanning two modalities and are listed under their primary modality; see Figure 2 for dual-counted modality totals. MID values reported in López-Blanco 2018, Elble 2017 and Rajan 2023 represent Minimum Detectable Changes or anchor-derived thresholds rather than formally derived Minimum Important Differences. ET, Essential Tremor; TRACE, Technology Readiness and Clinical Evidence framework; MID, Minimum Important Difference; PRO, Patient-Reported Outcome; MDC, Minimum Detectable Change; TAPS, Transcutaneous Afferent Patterned Stimulation; QUEST, Quality of Life in Essential Tremor Questionnaire; VHI, Voice Handicap Index; CRST-C, Clinical Rating Scale for Tremor Part C (patient self-report); IMU, inertial measurement unit; EMG, electromyography.

## Discussion

This scoping review maps 165 studies of digital tremor biomarkers in ET against a novel five-tier validation framework. The principal finding is that the field has generated a technically credible body of Tier 2 construct validity evidence, however the ecological, interpretability, and patient-centred evidence required to deploy any of these metrics as clinical trial endpoints is largely absent.

The included studies are heterogeneous by design, as expected in a scoping review mapping a broad technological landscape. Twenty three studies enrolled ET patients primarily as a comparator group for another disorder, 35 included ET within mixed-diagnosis cohorts, and one entered via the repeated-measures exception (Table 1). Diagnostic criteria varied: most recent studies applied or referenced the 2018 MDS consensus classification, but older studies frequently relied on unspecified clinical diagnosis, predating the formal distinction between ET and ET-plus. This heterogeneity does not undermine the principal finding, the concentration of evidence at Tier 2 and the near-absence of ecological validation is consistent across all study subgroups, but it does mean that pooled prevalence estimates for specific validation features should be interpreted with caution, and that future studies should specify which consensus criteria were applied.

**Figure 4:**
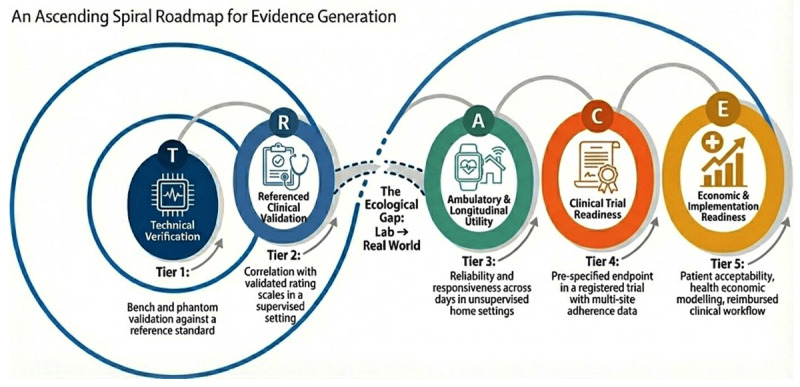
The TRACE Framework — A Five-Tier Maturity Roadmap for Digital Biomarkers in Neurological Disease. The TRACE framework provides a structured pathway for evaluating the validation maturity of digital biomarkers, progressing from controlled laboratory settings (centre) to real-world clinical implementation (outer loop). Each tier represents a distinct and cumulative evidence threshold: **T**echnical Verification (Tier 1) establishes sensor accuracy against a reference standard in bench or phantom testing; **R**eferenced Clinical Validation (Tier 2) demonstrates correlation with validated rating scales or diagnostic group discrimination in a supervised clinical setting; **A**mbulatory and Longitudinal Utility (Tier 3) requires reliability, stability, and responsiveness to change demonstrated across separate calendar days in unsupervised home or ambulatory settings; **C**linical Trial Readiness (Tier 4) requires the digital metric to be pre-specified as a primary or secondary endpoint in a registered trial, with operational delivery demonstrated through adherence and data completeness reporting; **E**conomic and Implementation Readiness (Tier 5) requires formal evidence of patient acceptability alongside at least one of: validated health economic modelling or demonstrated integration into a reimbursed clinical workflow. The dashed segment between Tiers 2 and 3 denotes the Ecological Gap, the critical translational hurdle at which most digital biomarkers stall, where laboratory validity does not predict real-world longitudinal performance. A study is assigned to the highest tier for which all core criteria are satisfied; tier assignment is not cumulative. TRACE adapts and extends the Digital Medicine Society (DiMe) V3 framework: Tier 1 corresponds to DiMe V3 Verification; Tiers 2 and 3 together deconstruct DiMe V3 Clinical Validation into snapshot and longitudinal components; Tiers 4 and 5 extend beyond DiMe V3 to encompass the regulatory and economic evidence required for clinical adoption. Tier 4 is operationalised in this review as clinical trial readiness rather than formal regulatory qualification, reflecting the current maturity of the field. Full tier definitions, core criteria, and decision rules are provided in Supplementary File 1.

Several frameworks exist for evaluating the maturity of digital health technologies, most notably the DiMe V3 model [23]. TRACE was developed for this review to address a gap: the need to formally separate single-session supervised validity from ambulatory and longitudinal utility, which remains the key translational bottleneck in many fields. By making the ecological transition an explicit, independently scorable tier rather than an implicit component of clinical validation, TRACE renders visible a gap that other frameworks obscure. The framework is visualised as an Archimedes spiral (Figure 4). Although derived for ET, TRACE was developed as a pragmatic framework for this review. Its tier criteria were designed to be transferable across sensing modalities and neurological conditions, and it may be applied prospectively as a design framework or retrospectively as an evidence mapping tool; however, formal external validation, inter-rater reliability testing, and application beyond ET remain priorities for future work.

Across 154 Tier 2 studies, digital tremor metrics correlate with established clinical rating scales and, in many cases, discriminate ET from healthy controls and other movement disorders under supervised conditions. Several platforms demonstrate sufficient precision to differentiate severity levels within ET cohorts, though this is not uniformly reported.

What Tier 2 cannot establish is whether these properties hold in the environment where clinical trials would deploy them. Tremor in ET is characteristically provoked by action and modulated by emotional state, fatigue, caffeine, and medication timing; its day-to-day variability in natural conditions is substantially greater than the within-session variance characterised in clinic studies. A device that correlates well with TETRAS during a standardised postural holding task in a neurology clinic may behave very differently when patients attempt the same task independently at home, without instruction, at varying times of day, and across weeks or months. The step from Tier 2 to Tier 3 is not an incremental refinement but a qualitative transition in what is being tested.

This Ecological Gap, the failure of clinic-validated metrics to transfer reliably to ambulatory real-world settings, is not unique to ET. The Mobilise-D consortium developed a validation roadmap for digital mobility outcomes across multiple chronic conditions that formally separates technical, real-world technical, and clinical validation as distinct sequential stages, motivated by the recognition that supervised laboratory performance cannot be assumed to predict real-world measurement behaviour [24]. The DiMe V3 framework is based on the same principle: analytical validation is necessary but insufficient; clinical validity must be demonstrated in the intended context of use [23].

For ET, this lesson carries a specific complication. In PD, the mobility impairments targeted by digital monitoring, gait slowing, bradykinesia, reduced stride variability, manifest continuously during ordinary daily movement, meaning that background recording over hours or days will naturally and repeatedly sample the clinically relevant signal. ET tremor is different in kind: postural and kinetic tremor requires the limb to be held against gravity or engaged in a voluntary task, and is largely absent during rest, quiet sitting, or low-demand movement. A wrist sensor worn throughout the day will therefore spend most of its recording time in a signal state that does not represent the tremor that constitutes the clinical target. Closing the ecological gap for ET digital biomarkers consequently requires either structured task protocols delivered at home, asking patients to perform a postural holding or drawing task at a specified time, or the development of algorithms capable of identifying and extracting task-relevant epochs from free-living signal streams. The structured task approach has been validated at scale only within the Cala Health TAPS platform, where Isaacson and colleagues and Dai and colleagues deployed repeated home-based postural hold assessments across two registered multi-site trials enrolling over 500 patients, with pre-specified digital endpoints and quantified adherence [21,37]; however, both studies are embedded within a therapeutic intervention programme rather than an independent biomarker validation context. Algorithm-based extraction of task-relevant epochs from free-living signal streams has not been validated at scale in ET, though Pulliam and colleagues demonstrated its feasibility alongside structured assessments in 20 patients across two calendar-separated days, finding that continuous waveform metrics correlated sufficiently with structured task scores to suggest epoch extraction as an alternative to prompted assessments [28].

No formally anchored minimum important difference exists for any ET digital biomarker. The three studies reporting MID-type values, Elble and Ellenbogen for digitised spirals, López-Blanco and colleagues for smartwatch gyroscopy, and Rajan and colleagues for tablet-derived mean deviation, all quantify measurement precision rather than patient-perceived meaningful change [38,41,43]. Elble and Ellenbogen’s MDC of 51% of baseline amplitude, for example, means tremor must roughly halve before a change exceeds measurement error [41]. Anchor-based MID derivation, requiring longitudinal data from patients crossing a clinically meaningful threshold alongside concurrent digital measurements, is methodologically straightforward but depends on the multi-visit, home-capable, PRO-integrated study design that remains largely absent from the current literature.

This matters directly for the regulatory pathway toward qualified drug development endpoints. Both the FDA’s Drug Development Tool (DDT) qualification programme and the EMA’s Qualification of Novel Methodologies process provide voluntary mechanisms through which a digital metric can be qualified for a specific context of use, but both require evidentiary packages extending well beyond analytical validation: clinical meaningfulness, minimum important difference estimation anchored to patient experience, and sensitivity to change in the intended context of use [44]. In the UK, software-based tremor analysis meeting the definition of a medical device falls under MHRA regulation as Software as a Medical Device. Across all three regulatory environments, the pathway from a metric that correlates with TETRAS in a clinical setting to one qualified as a drug development tool requires ecological validation and clinically anchored evidence of meaningful change, neither of which the current ET literature can provide for any modality. To date, no ET digital biomarker has achieved formal DDT qualification under either the FDA or EMA programmes [44]. The TAPS platform, deployed in the only two studies assigned Tier 4, demonstrates that clinical trial readiness is achievable for an ET digital endpoint; across two registered trials enrolling over 500 patients, tremor power was pre-specified as a digital outcome measure with multi-site recruitment, quantified adherence, and longitudinal tracking [21,37]. However, both studies are embedded within a therapeutic intervention programme rather than an independent biomarker validation context, reflecting the needs of the TAPS trial ecosystem rather than the broader qualification work, including ecological validation and patient-anchored MID estimation, required for formal regulatory qualification as a reusable drug development tool. The measurement protocol, repeated home-based accelerometry with structured postural holds and cloud-based data capture, is separable from the therapeutic stimulation component and could be deployed independently as a digital endpoint in trials of other ET interventions.

The longer-term question, whether validated digital tremor metrics could one day guide treatment decisions in routine practice, as MRI lesion burden has in multiple sclerosis, is the question Tier 5 is designed to address [45]. No study in this review approached that threshold; economic and implementation readiness remains contingent on first bridging the ecological gap that separates most modalities from Tier 3.

The concentration of digital biomarker evidence in upper-limb postural and kinetic tremor reflects the primary diagnostic criterion for classical ET and the most tractable target for existing sensor technologies. However, ET is increasingly understood as a heterogeneous condition: head tremor is a recognised feature of the full syndrome, voice tremor affects a substantial minority of patients [46], and gait and balance impairment, while not diagnostic, are associated features that worsen with disease duration and may be exacerbated by stereotactic thalamic interventions [47]. The ET-plus classification formalised in the 2018 MDS consensus criteria encompasses patients with additional neurological signs including mild ataxia, dystonia, and rest tremor, and these patients may present to clinical trials in increasing numbers as eligibility criteria broaden [3].

If digital biomarker endpoints are to serve trials targeting the breadth of the ET phenotype, including ablative procedures and neuromodulation, validated metrics for non-upper-limb domains will be required. Six voice studies now demonstrate that vocal tremor is measurable and discriminable using accessible acoustic analysis equipment, with Suppa and colleagues providing the broadest validation profile including treatment responsiveness and PRO correlation (Tier 2+); however, no voice study has deployed outside the clinic, and home recording data represent a tractable next step [42]. The four gait studies are limited to single-session designs, and none characterises ET gait in its own right (Table 1). Dedicated ET gait and balance monitoring, drawing on the Mobilise-D method of seven-day home deployment, would address both the phenotypic coverage gap and the ecological validity deficit simultaneously [24,48]. The diagnostic criteria heterogeneity observed across studies, where enrolment criteria are frequently underspecified, is a related concern: pooling data across studies that may have included ET, ET-plus, and dystonic tremor without differentiation limits both the interpretation of existing findings and the generalisability of any future MID derivation.

Translating the current evidence base into trial-ready endpoints requires a sequenced programme of work. For IMUs, the priority is extending ambulatory Tier 3 evidence beyond the TAPS ecosystem, which accounts for five of eight Tier 3 studies. Feasibility studies using commercially available, clinically validated platforms such as Kinesia (Great Lakes NeuroTechnologies) and the Clario Opal, both with established clinical trial infrastructure, deploying structured home task protocols with concurrent clinical rating scales and PRO instruments across a minimum of two calendar-separated sessions, would establish whether the ambulatory findings from the TAPS platform generalise to independent devices. For digitised handwriting, no study has deployed in the home setting; the next step is a home deployment feasibility study incorporating a familiarisation session, as recommended by the Elble practice effect data, followed by multi-visit MDC reassessment and an anchor-based MID derivation sub-study [26]. For computer vision, independent replication of the Baek postal drawing approach in a prospective ET cohort with a standardised analysis pipeline would establish whether this method is generalisable [36]; the absence of a commercially validated contactless platform represents an additional barrier to near-term trial deployment not faced by the IMU or handwriting modalities. For sEMG, all ten studies are lab-based and single-session; feasibility of home-based EMG recording for ET has not been explored.

Across all modalities, three cross-cutting requirements apply: standardised diagnostic criteria reporting, specifying whether MDS 2018 consensus criteria were applied and whether ET-plus patients were included [3]; incorporation of PRO instruments (QUEST, TETRAS-ADL, Voice Handicap Index) as standard in all Tier 2+ studies to anchor MID derivation and ensure patient-centred interpretability; and adoption of a consensus minimum assessment dataset, as proposed by Varghese and colleagues [49]. Prospective application of the TRACE framework within longitudinal observational cohorts, using tier criteria as pre-specified design requirements rather than post-hoc labels, would provide a structured pathway toward the ecological and interpretability evidence currently absent from the literature.

Several limitations of this review should be noted. As a scoping review, formal critical appraisal was not performed; TRACE tier assignment captures validation maturity, not methodological rigour, and a higher tier should not be interpreted as indicating superior study quality. The modality imbalance, 114 of 165 studies using wearable IMUs, means field interpretation is disproportionately informed by a single sensor class. Extraction from mixed-population studies may have introduced imprecision in ET-specific estimates, and unpublished evidence could alter some tier assignments. Finally, publication bias towards positive construct validity findings is likely, and the absence of null validation studies may overstate the validity of Tier 2 evidence for individual platforms.

One hundred and sixty-five studies across six sensing modalities confirm that digital tremor measurement in ET is technically feasible and clinically anchored in supervised settings, but the ecological, patient-centred, and regulatory-ready evidence required for trial deployment is absent outside the Cala Health TAPS platform [21,37]. What the field requires is not further construct validity accumulation in single-session clinic studies, but deliberate investment in home deployment, MID derivation, and PRO integration across independent platforms. The TRACE framework, described in full in Supplementary File 1, provides a potential transferable tool for other chronic neurological conditions where these validation gaps are likely to recur.

## Artificial Intelligence Disclosure

Artificial intelligence tools (Claude, Anthropic) were used to assist with manuscript preparation and editing. Additionally, Gemini 3.1 Flash Image (Nano Banana 2) was used to co-generate the visual components of Figure 4. No other figures were produced or modified using AI tools. All content was reviewed, verified, and approved by all authors. AI tools were not used to generate or alter primary data.

## Additional Files

The additional files for this article can be found as follows:

10.5334/tohm.1205.s1 Supplementary File 1. The TRACE Maturity Framework for Digital Biomarkers in Neurological Disease.

10.5334/tohm.1205.s2 Supplementary File 2. Electronic Search Strategies.

10.5334/tohm.1205.s3 Supplementary File 3. TRACE-ET Data Charting Instrument.
