# Supplementary material for: Essential Tremor and Digital Biomarkers: A Scoping Review Using the TRACE Framework to Map Readiness for Clinical Trials and Routine Practice
Source: Tremor Other Hyperkinet Mov (N Y). 2026 Jun 8;16:39. doi: 10.5334/tohm.1205 (PMC13262646; doi:10.5334/tohm.1205)
Supplement: Supplementary File 1. — The TRACE Maturity Framework for Digital Biomarkers in Neurological Disease. [file tohm-16-1-1205-s1.pdf]

Supplementary File 1

The TRACE Maturity Framework for Digital Biomarkers in Neurological Disease

Full Tier Definitions, Core Criteria, Tier 2/3 Decision Flowchart, and Boundary Case Rules

ESSENTIAL TREMOR AND DIGITAL BIOMARKERS: A SCOPING REVIEW USING THE TRACE FRAMEWORK TO MAP READINESS FOR CLINICAL TRIALS AND ROUTINE PRACTICE| PRISMA-SCR ALIGNED | LEDINGHAM, MACEROLLO, PAVESE

This document sets out the tier definitions, core criteria, and decision rules for the TRACE Validation Framework as applied in this scoping review. It should be read alongside the main methods section and Supplementary File 2 (the data charting instrument).

Rationale for Developing TRACE

The DiMe V3 model (Verification, Analytical Validation, Clinical Validation), published by the Digital Medicine Society, provides a widely adopted foundation for evaluating digital health technologies but groups all clinical evidence into a single validation stage(1). In practice, a sensor that correlates with a clinical rating scale in a supervised clinic session has demonstrated something qualitatively different from one that captures intra-individual tremor fluctuations reliably over days or weeks in a patient's home. The existing framework does not make this distinction visible.

TRACE was developed to address this gap. It splits the DiMe V3 Clinical Validation phase into two tiers, Referenced clinical validity (Tier 2), covering supervised single-session evidence, and Ambulatory and longitudinal utility (Tier 3), requiring unsupervised home data across separate calendar days, and extends the pathway to include the operational evidence needed for clinical trial deployment (Tier 4) and the health economic and patient-centred evidence needed for clinical practice adoption (Tier 5). The framework was calibrated to the current maturity of the ET digital biomarker field; it is a roadmap, not a regulatory compliance checklist.

Operationalisation Note — Tier 4

Tier 4 in this review is operationalised as clinical trial readiness rather than formal regulatory qualification (FDA Drug Development Tool qualification, EMA Qualification of Novel Methodologies, or equivalent). No ET digital biomarker has yet reached formal regulatory qualification, so the operationally relevant question for this field is whether a digital metric is ready for deployment as a pre-specified endpoint in a registered interventional trial. Formal regulatory qualification, where achieved, constitutes the strongest possible evidence at Tier 4 but is not required for assignment. Critically, Tier 4 reflects operational deployment capacity, whether a digital metric can be delivered reliably across sites with adequate adherence and data completeness, not the therapeutic outcome of the trial in which it was deployed; a metric used as a pre-specified endpoint in a trial that fails to demonstrate treatment efficacy remains Tier 4 provided the measurement infrastructure performed as intended. This operationalisation is noted in the main methods section and in Supplementary File 2. Reviewers applying TRACE in fields where regulatory qualification has been achieved should reinstate formal qualification criteria as the primary Tier 4 standard.

General Assignment Principles

Each study is assigned to the highest tier for which all core criteria are satisfied. Assignment reflects the most advanced validation domain demonstrated, not cumulative fulfilment of every criterion at lower tiers. Where ambiguity remained after applying the decision rules, the conservative (lower tier) assignment was applied and documented with a rationale note in Field 12 of the charting instrument. Borderline cases were reviewed against at least two other papers at the same boundary to ensure consistency.

Tier 2+ is used as an internal annotation for multi-visit clinic studies (separate calendar days, clinic only, no home component). These are presented as Tier 2 in all published outputs but flagged in the master data file to distinguish them from single-session Tier 2 studies for sensitivity analysis.

Part A — Five-Tier Definitions Table

Table 1 defines the five TRACE tiers. For each tier the table provides: core criteria that must all be met for assignment; distinguishing features and edge cases separating it from adjacent tiers; the current status of that tier in the ET literature; and a worked example from the reviewed studies.

Tier	Label	Core Criteria (ALL must be met)	Distinguishing Features & Edge Cases	ET Field Status	Worked Example
Tier 1	Technical Verification	Bench or phantom testing only. No ET patients required. Device accuracy validated against a mechanical or electrical reference standard (e.g., motor-driven shaker, calibrated signal generator). Accuracy, precision, linearity, and noise characteristics reported.	No biological subjects, sensor performance only. Pure Tier 1 papers without ET patient data are EXCLUDED from this review. Papers combining bench validation WITH ET patient data are INCLUDED and assigned to the highest tier achieved in the patient component; the Tier 1 bench component is noted as a flag.	Rarely reported in isolation in the ET literature. Most sensor validation is performed as a component within Tier 2 studies rather than as standalone bench studies.	Accelerometer validated on a motor-driven mechanical shaker platform. Gyroscope calibration study using a precision rotating stage.
Tier 2	Referenced Clinical Validity	ET patients included (ET n ≥10 or repeated-measures exception). Digital metric reported in a supervised clinical or laboratory setting. At least ONE of the following demonstrated: (a) correlation with a validated clinical scale (TETRAS, FTM-TRS, CRST, Bain-Findley) or clinician severity	Single session or single calendar day only. Lab or clinic setting. Pre/post medication, DBS ON/OFF, or ethanol challenge within the same visit = Tier 2 Immediate Responsivenes s, not Tier 3. Multi-visit clinic data (separate calendar days, clinic only) = Tier 2+ (flagged), does not elevate to	The dominant tier in the current ET literature. The majority of digitising tablet, IMU, and EMG studies in ET achieve Tier 2. Represents the evidence base from which the field must progress.	Cross-sectional spiral analysis correlated with TETRAS (r=0.72) in a single clinic session (Mostile 2010). Pre/post propranolol tremor amplitude in a single clinic visit. DBS ON vs OFF within one appointment.(2)

		estimate; (b) discrimination of ET from healthy controls, PD, or another disorder; (c) test-retest reliability within a single clinic visit; (d) immediate responsiveness to a within-session intervention (medication, DBS ON/OFF, ethanol challenge).	Tier 3 without a home/ambulatory component.		
Tier 3	Ambulatory & Longitudinal Utility	ALL THREE of the following must be met: (a) Temporal separation, measurements on at least two separate calendar days with the same participants; (b) Home or ambulatory setting, at least one measurement occasion collected outside a supervised clinical visit, with patients using the device independently; (c) Temporal performance evidence, at least one of: reliability statistics across days (ICC, CV, Bland-Altman), sensitivity to change across separated time points, or	Multi-visit clinic data alone (even across many clinic days) does NOT qualify — the ambulatory home setting criterion must be met. Small ET n (n<15) does not disqualify but should be flagged as feasibility-grade Tier 3. Therapeutic device studies (e.g., TAPS wristband) with home tremor metrics qualify and are flagged as therapeutic context.	The critical validation gap in the ET literature. Very few studies achieve Tier 3. Those that do are primarily wearable IMU studies. Digitised handwriting, surface EMG, and computer vision technologies are largely absent at this tier.	Home accelerometry across 7 days with day-to-day ICC=0.77–0.95 (Pulliam 2014). Smartwatch continuous monitoring across 26 hours of daily life, r=0.87 day-to-day (Zheng 2017)(3).

		longitudinal tremor tracking reported.			
Tier 4	Clinical Trial Readiness	ALL of the following must be present: (a) The digital metric was pre-specified as a primary or secondary endpoint in a registered clinical trial (ClinicalTrials.gov or equivalent national registry), with operational delivery demonstrated through adherence and data completeness reporting; multi-site deployment across ≥3 independent sites satisfies the operational delivery criterion but does not substitute for pre-registration; (b) A defined measurement context exists: task, posture, duration, and algorithm are specified; (c) The study design is interventional or prospective. Note: Tier 4 in this review is operationalised as clinical trial readiness rather than formal regulatory qualification (FDA DDT, EMA	Multi-site data and adherence reporting from a Tier 3 study WITHOUT pre-registration constitutes a Tier 4 signal — flag in F24 but retain the assigned tier. Formal FDA DDT or EMA qualification constitutes the strongest possible evidence at this tier but is not required.	Rare in the ET literature. Only one study in this review reaches Tier 4 (Isaacson 2020 — PROSPECT trial: TAPS wristband pre-specified as primary endpoint across 16 sites in a registered interventional trial with adherence reporting)(4). Represents the immediate target for the most advanced modalities.	PROSPECT trial (NCT03597100): TAPS wristband tremor power pre-specified as primary endpoint; 16 sites; 193 patients; 3-month prospective design; adherence reported (21,806 sessions); defined measurement context (postural task, 30s, Cala Health algorithm) (Isaacson 2020)(4).

		qualification advice). This reflects the current maturity of the ET field			
Tier 5	Economic & Implementation Readiness	Evidence bearing on whether the digital biomarker can be sustainably integrated into clinical practice or long-term trials from the perspective of patients and healthcare systems. Assign if the study satisfies the following: (a) REQUIRED — formal patient acceptability or usability data from a validated questionnaire or structured patient survey, or patient burden and adherence data reported from the patient perspective (not just session completion rates); AND (b) AT LEAST ONE OF — health economic modelling or cost-effectiveness analysis; OR demonstrated integration into a routine clinical workflow or reimbursement pathway. NICE, ICER, or IQWiG appraisal constitutes the	Absence of Tier 5 evidence across the ET literature is itself a key finding of this review and should be reported explicitly in the results and discussion. The PKG (Personal KinetiGraph) in Parkinson's disease represents the aspirational precedent, a wearable device that has traversed the full validation pathway to reimbursement in some health systems.	Effectively absent from the current ET digital biomarker literature. No study in this review achieves Tier 5. Tier 5 evidence is discussed conceptually in some papers but no formal health economic modelling or validated patient acceptability instrument data specific to ET digital biomarkers has been published.	Aspirational precedent (not in current ET corpus): PKG (Personal KinetiGraph) in Parkinson's disease, reimbursed in Australia for clinical tremor and dyskinesia monitoring; NICE health technology appraisal of a tremor monitoring device. No ET example currently exists.

		strongest evidence for criterion (b) but is not required.			

Part B — Tier 2/3 Boundary Decision Flowchart

The Tier 2/3 boundary is the most frequently ambiguous classification decision in the framework and the most common source of over-assignment to Tier 3. Apply the three questions below sequentially to every study that reaches at least Tier 2. Stop as soon as a 'No' is reached. Worked examples of how these questions were applied during extraction are provided in Supplementary File 2, Part D.

**Step**	**Question**	**Criteria**	**Decision**
Q1	Calendar day separation	Were measurements taken on at least two separate calendar days with the same participants? Note: multiple time points, tasks, or measurement occasions within the same day (including across several hours within a single clinic session) do NOT constitute calendar day separation.	NO → Assign Tier 2. Stop. Do not proceed to Q2. YES → Proceed to Q2. This is the threshold criterion. It cannot be bypassed.
Q2	Ambulatory / home setting	Were any measurements taken in a home or ambulatory setting, outside a supervised clinical or laboratory visit, with patients using the device independently? Note: supervised home visits by a researcher (the researcher travels to the patient's home) are classified as lab-clinic, not home, the ecological validity advantage derives from absence of supervision, not from location alone.	NO (clinic only, even across multiple visit days) → Assign Tier 2 with multi-visit clinic flag. Stop. Do not proceed to Q3. YES → Proceed to Q3.
Q3	Temporal performanc e evidence	Is there evidence of temporal performance across those separated home or ambulatory time points? At least ONE of the following must be present: (a) Reliability statistics across days: ICC, CV, Bland-Altman limits of agreement, Pearson or Spearman correlation across sessions (b) Responsiveness to change: demonstrated sensitivity to treatment effect or natural disease variation across the separated time points (c) Longitudinal tracking: tremor metrics charted, modelled, or summarised across ≥2 home sessions over time	At least ONE criterion met → Assign Tier 3. None met (home device used but no temporal performance data reported across days) → Assign Tier 2 with flag 'home deployment, no temporal performance data reported'. The device reached the home, but the validation evidence did not.

Part C — Tier-Specific Boundary Case Rules

Table 3 provides decisions for the most common boundary and edge cases encountered during framework development and tested against the included literature. These serve as precedents: when a structurally equivalent scenario arises, the documented decision should be applied directly.

Scenario	Tier Assigned	Rationale	Decision Rule
Pre/post medication (e.g., propranolol) or ethanol challenge within a single clinic visit Ref: Haubenberger 2011(5)	Tier 2 (Immediate Responsiveness)	Both measurements occur within a single clinical episode on the same calendar day. No temporal separation between visits; no home element. The ethanol challenge paradigm with 6 time points across 75 minutes is a single-session study regardless of the number of time points.	Single calendar day = maximum Tier 2, regardless of the number of measurement occasions or interventions within that day.
DBS stimulation ON vs OFF tested within one clinic visit. General rule; exemplified across multiple IMU and EMG studies in this review	Tier 2 (Immediate Responsiveness)	Two physiological states tested sequentially within a single clinical episode. No calendar day separation; no ambulatory element.	As above. The number of physiological states tested does not affect tier assignment, only temporal and contextual criteria matter.
Pre-operative, intra-operative, and immediate post-operative measurement on the same procedure day Ref: Tam 2017 (MRgFUS)(6)	Tier 2 (Immediate Responsiveness)	Multiple time points all within a single clinical episode (the surgical procedure day). Example: MRgFUS spiral drawing pre/intra/post on the same day.	Calendar day is the temporal unit. All measurements same day = Tier 2, regardless of clinical context (including surgical context).
Test-retest reliability across two separate clinic visits on different calendar days, clinic setting only Ref: Elble 2017(7)	Tier 2+ (multi-visit clinic flag)	Calendar day separation is present (Q1 = Yes) but no home or ambulatory component (Q2 = No). Temporal separation alone is insufficient, ecological validity requires independent unsupervised data collection.	Apply multi-visit clinic flag. Present as Tier 2 in all published outputs. Do not elevate to Tier 3.
Take-home device used across two separate days with reliability statistics reported, small ET n (n<15) Ref: General Tier 3 feasibility pattern; exemplified by Pulliam 2014(8)	Tier 3 (feasibility-grade flag)	All three Tier 3 criteria met: calendar day separation (Q1 = Yes), home/ambulatory setting (Q2 = Yes), day-to-day reliability statistics reported (Q3 = Yes). Small n is a quality descriptor, not a tier disqualifier.	Assign Tier 3. Apply feasibility-grade Tier 3 flag (ET n<15). Note sample size adequacy in F27.
Therapeutic device study (e.g., TAPS	Tier 3 or Tier 4 depending on	Primary purpose is therapeutic but digital tremor metric is	Include. Apply therapeutic

wristband) with home tremor metrics collected over weeks/months Ref: Isaacson 2020(4)	registration status	extracted and reported across home sessions. If pre-registered with pre-specified tremor endpoint and multi-site, Tier 4 may apply. Therapeutic context does not preclude high-tier assignment.	context flag. Assign tier based on highest evidence criteria met in the tremor measurement component, not the therapeutic intent.
Multi-site study with adherence reporting from a Tier 3 design, but NOT pre-registered with a specified tremor endpoint Ref: General Tier 4 signal rule	Tier 3 (retain) Tier 4 signal flag	Multi-site data and adherence reporting are present but the pre-registration and defined measurement context criteria for Tier 4 are not fully met. These studies represent the strongest candidates for Tier 4 promotion in future work.	Retain Tier 3 as the assigned tier. Apply Tier 4 signal flag in F24 for sensitivity analysis tracking.

Part D — Tier 2+ and Sensitivity Analysis Notes

Tier 2+ (Multi-visit Clinic)

Studies collecting data across two or more separate calendar days in a supervised clinical setting, but with no home or ambulatory component, are assigned Tier 2 and flagged as multi-visit clinic. They appear as Tier 2 in all published outputs. In the master data file they are distinguished from single-session Tier 2 studies to support two sensitivity analyses: first, whether the construct validity evidence base changes when multi-visit clinic studies are analysed separately; and second, what proportion of Tier 2 studies have reliability data (from multi-visit designs) versus construct or discrimination validity only (from single-session designs). This stratification is reported in the results.

Feasibility-grade Tier 3

Tier 3 studies with ET $n<15$ are assigned Tier 3 but flagged as feasibility-grade. The small sample limits the statistical precision and generalisability of the ambulatory and longitudinal evidence. These studies are included in the Tier 3 evidence base but distinguished in the results narrative from those with larger samples.

Tier 4 Signal

Studies demonstrating multi-site data collection or formal adherence reporting within a Tier 3 design, but without pre-registration or a fully defined measurement context, are retained at Tier 3 and flagged as Tier 4 signal. These represent the strongest candidates for full Tier 4 evidence in future work and are highlighted in the results accordingly.

References

1. Goldsack JC, Coravos A, Bakker JP, Bent B, Dowling AV, Fitzer-Attas C, et al. Verification, analytical validation, and clinical validation (V3): the foundation of determining fit-for-purpose for Biometric Monitoring Technologies (BioMeTs). npj Digital Medicine. 2020;3(1):55.
2. Mostile G, Giuffrida JP, Adam OR, Davidson A, Jankovic J. Correlation between Kinesia system assessments and clinical tremor scores in patients with essential tremor. Movement Disorders. 2010;25(12):1938-43.
3. Zheng X, Vieira Campos A, Ordieres-Meré J, Balseiro J, Labrador Marcos S, Aladro Y. Continuous Monitoring of Essential Tremor Using a Portable System Based on Smartwatch. Front Neurol. 2017;8:96.
4. Isaacson SH, Peckham E, Tse W, Waln O, Way C, Petrossian MT, et al. Prospective Home-use Study on Non-invasive Neuromodulation Therapy for Essential Tremor. Tremor Other Hyperkinet Mov (N Y). 2020;10:29.
5. Haubenberger D, Kalowitz D, Nahab FB, Toro C, Ippolito D, Luckenbaugh DA, et al. Validation of digital spiral analysis as outcome parameter for clinical trials in essential tremor. Mov Disord. 2011;26(11):2073-80.
6. Tam F, Huang Y, Schwartz ML, Schweizer TA, Hynynen K, Graham SJ. A computerized tablet system for evaluating treatment of essential tremor by magnetic resonance guided focused ultrasound. BMC Neurol. 2017;17(1):74.
7. Elble RJ, Ellenbogen A. Digitizing Tablet and Fahn-Tolosa-Marín Ratings of Archimedes Spirals have Comparable Minimum Detectable Change in Essential Tremor. Tremor Other Hyperkinet Mov (N Y). 2017;7:481.
8. Pulliam CL, Eichenseer SR, Goetz CG, Waln O, Hunter CB, Jankovic J, et al. Continuous in-home monitoring of essential tremor. Parkinsonism Relat Disord. 2014;20(1):37-40.

Citation

When using or adapting this instrument, please cite: Ledingham D, Macerollo A, Pavese N. Essential Tremor and Digital Biomarkers: A Scoping Review Using the TRACE Framework to Map Readiness for Clinical Trials and Routine Practice. Tremor and Hyperkinetic Disorders. [Year]. Supplementary File 2: TRACE-ET Data Charting Instrument.

The TRACE framework is intended to be a living tool. Researchers applying it in other movement disorder contexts are encouraged to document any adaptations to the tier definitions, particularly for Tier 4 (where regulatory context may differ) and Tier 3 (where the ambulatory criterion may need specification for conditions with different monitoring requirements than tremor).
