# Supplementary material for: Essential Tremor and Digital Biomarkers: A Scoping Review Using the TRACE Framework to Map Readiness for Clinical Trials and Routine Practice
Source: Tremor Other Hyperkinet Mov (N Y). 2026 Jun 8;16:39. doi: 10.5334/tohm.1205 (PMC13262646; doi:10.5334/tohm.1205)
Supplement: Supplementary File 2. — Electronic Search Strategies. [file tohm-16-1-1205-s2.pdf]

Supplementary File 2 — Electronic Search Strategies

Original Search

A structured search was conducted across four electronic databases: PubMed, Web of Science, Scopus, and IEEE Xplore. IEEE Xplore was included to capture engineering and signal processing literature that is underrepresented in biomedical databases. The same search string was applied across all four databases. Search terms were grouped into two conceptual domains, Essential Tremor and digital assessment technologies, and combined using Boolean operators. Terms relating to clinical relevance, treatment response, or biomarker correlation were deliberately excluded from the search string to avoid overly restrictive filtering; these aspects were assessed during full-text screening.

The search string applied to all four databases was:

("Essential Tremor" OR "ET") AND ("digital biomarkers" OR "wearable devices" OR "IMU" OR "smartphone" OR "spiral analysis")

Searches were limited to articles published in English. No date restriction was applied at the search stage; publications prior to 1 January 2000 were excluded at the screening stage and are documented in the PRISMA flow diagram. The final database search was executed on 8 September 2025. Following database searching, the reference lists of all included studies and relevant review articles were hand-searched to identify additional eligible publications.

Supplementary Search

Following peer review, a supplementary electronic database search was conducted to ensure comprehensive capture of studies using standalone sensor-specific terminology that was not included in the original search string. In particular, the reviewer noted that studies using accelerometry or surface electromyography as digital outcome measures in essential tremor would not be captured by the original technology-platform terms (e.g. “wearable devices,” “IMU”). The supplementary search therefore incorporated MeSH terms and free-text terms for accelerometry, electromyography, gyroscopy, goniometry, computer vision, and acoustic voice analysis. It was applied across the same four bibliographic databases as the original search. Results were deduplicated internally (across the four supplementary database exports), then against the original search yield, before title and abstract screening using the same eligibility criteria.

Supplementary Search database search strings

PubMed

("Essential Tremor"[MeSH] OR "Essential Tremor"[tiab])

AND

("Accelerometry"[MeSH] OR acceleromet*[tiab] OR accelerometer*[tiab]

OR "Electromyography"[MeSH] OR electromyograph*[tiab]

OR "surface EMG"[tiab] OR "sEMG"[tiab]

OR gyroscop*[tiab] OR goniometer*[tiab] OR goniometr*[tiab]

OR "motion sensor"[tiab] OR "motion sensors"[tiab]

OR "inertial sensor"[tiab] OR "inertial sensors"[tiab]

OR "tremor monitor*"[tiab]

OR "computer vision"[tiab] OR "pose estimation"[tiab]

OR "video analysis"[tiab]

OR "voice analysis"[tiab] OR "acoustic analysis"[tiab])

AND 2000/01/01:2026/04/27[dp]

AND English[la]

Notes: MeSH terms used for Essential Tremor, Accelerometry, and Electromyography. Title/abstract field tags [tiab] for all free-text terms. "ET" excluded as a standalone term due to non-specific matching.

Web of Science (Core Collection)

TS=("Essential Tremor")

AND

TS=(acceleromet* OR accelerometer* OR electromyograph*

OR "surface EMG" OR "sEMG"

OR gyroscop* OR goniometer* OR goniometr*

OR "motion sensor" OR "motion sensors"

OR "inertial sensor" OR "inertial sensors"

OR "tremor monitor*"

OR "computer vision" OR "pose estimation"

OR "video analysis"

OR "voice analysis" OR "acoustic analysis")

Filters applied via interface: Publication Years 2000–2025; Language = English; Database = Web of Science Core Collection.

Scopus

TITLE-ABS-KEY("Essential Tremor")

AND

TITLE-ABS-KEY(acceleromet* OR accelerometer* OR electromyograph*

OR "surface EMG" OR "sEMG"

OR gyroscop* OR goniometer* OR goniometr*

OR "motion sensor" OR "motion sensors"

OR "inertial sensor" OR "inertial sensors"

OR "tremor monitor*"

OR "computer vision" OR "pose estimation"

OR "video analysis"

OR "voice analysis" OR "acoustic analysis")

AND PUBYEAR > 1999

AND LANGUAGE(english)

IEEE Xplore

Two searches were conducted due to IEEE Xplore query length limitations and combined manually. Both searches were restricted to the Abstract field to avoid full-text matching noise; an initial unrestricted search returned over 23,000 records, the majority of which referenced essential tremor incidentally in body text rather than as a study focus.

Search A:

("Abstract":"Essential Tremor")

AND

("Abstract":acceleromet* OR "Abstract":accelerometer*

OR "Abstract":electromyograph* OR "Abstract":"surface EMG"

OR "Abstract":"sEMG"

OR "Abstract":gyroscop* OR "Abstract":goniometer*)

Search B:

("Abstract":"Essential Tremor")

AND

("Abstract":"motion sensor" OR "Abstract":"motion sensors"

OR "Abstract":"inertial sensor" OR "Abstract":"inertial sensors"

OR "Abstract":"tremor monitor*"

OR "Abstract":"computer vision" OR "Abstract":"pose estimation"

OR "Abstract":"video analysis"

OR "Abstract":"voice analysis" OR "Abstract":"acoustic analysis")

Filters applied via interface: Year range 2000–2025; Content Type = Conferences + Journals.

Handling of Duplicate Datasets

Where the original and supplementary searches identified studies by the same first author using the same dataset published as both a conference paper and a subsequent journal article, the journal-published version was retained and the conference paper excluded. These replacements are documented in the PRISMA flow diagram as full-text exclusions from the original search arm (“superseded by journal version identified in supplementary search”).
