# Supplementary material for: Essential Tremor and Digital Biomarkers: A Scoping Review Using the TRACE Framework to Map Readiness for Clinical Trials and Routine Practice
Source: Tremor Other Hyperkinet Mov (N Y). 2026 Jun 8;16:39. doi: 10.5334/tohm.1205 (PMC13262646; doi:10.5334/tohm.1205)
Supplement: Supplementary File 3. — TRACE-ET Data Charting Instrument. [file tohm-16-1-1205-s3.pdf]

Supplementary File 3

TRACE-ET Data Charting Instrument

A Reproducible Data Charting Tool for Scoping Reviews of Digital Biomarkers in Movement Disorders

ESSENTIAL TREMOR AND DIGITAL BIOMARKERS: A SCOPING REVIEW USING THE TRACE FRAMEWORK TO MAP READINESS FOR CLINICAL TRIALS AND ROUTINE PRACTICE| PRISMA-SCR ALIGNED | LEDINGHAM, MACEROLLO, PAVESE

This instrument was developed for a PRISMA-ScR scoping review of digital biomarkers in Essential Tremor (ET)(1). It operationalises the TRACE Validation Framework, Technical verification (Tier 1), Referenced clinical validity (Tier 2), Ambulatory and longitudinal utility (Tier 3), Clinical trial readiness (Tier 4), and Economic and implementation readiness (Tier 5), and can be adapted for use in other movement disorders or chronic neurological conditions.

Twenty-seven fields are applied to every included study, grouped into seven sections: study identity (Fields 1–6), technology (7–10), TRACE tier evidence (11–13), construct validity (14–15), responsiveness and longitudinal evidence (16–19), regulatory and interpretability signals (20–23), and flags, notes, modality, and sample size (24–27 plus a narrative summary). One modality-specific sub-field (Field 26) is completed according to the sensing technology used. Where information is not reported in the source paper, record 'Not reported' (NR) rather than leaving the field blank; unreported data points are informative in a field where many validation gaps reflect omission rather than negative findings.

The instrument has four parts:

- **Part A** — the 27-field reference table.
- **Part B** — decision guidance for fields requiring interpretive judgement, including the study design disambiguation rules and the Tier 2/3 boundary flowchart (reproduced here for standalone use; the definitive version with full boundary case rules is in Supplementary File 3).
- **Part C** — extraction flag definitions.
- **Part D** — worked examples from boundary cases resolved during this review, included as precedents for future reviewers.

Reuse and adaptation. The instrument can be adapted for scoping reviews in other movement disorders. When doing so, update: 1) the TRACE tier definitions in Supplementary File 3 to reflect the maturity of the target field; 2) the modality categories in Field 26; and 3) the clinical anchor categories in Field 13 for the relevant rating scales.

Part A — The Charting Instrument

Field	Field Name	Response Format	Definition
**Section 1: Study Identity (Fields 1–6)**			
F1	First Author, Year	Free text	Surname of first author and four-digit publication year. Use journal publication year, not online-first date.
F2	Journal / Conference	Free text	Full journal name as published. Note if conference proceedings rather than peer-reviewed journal.
F3	Country	Free text	Country of primary data collection institution. If different from author affiliation country, record the data collection country.
F4	Study Design	Categorical — single-session / test-retest / multi-visit clinic / longitudinal / feasibility / RCT / other (specify)	Primary temporal structure of data collection. This is the most common source of misclassification — see Part B for full disambiguation rules and worked examples.
F5	ET n	Integer; note if subgroup	Number of ET participants only. If ET is a subgroup within a mixed-diagnosis study, record ET n and total study n separately (e.g., 'ET n=18, total n=45'). If a bench validation component exists alongside patient data, note this explicitly.
F6	ET Diagnostic Criteria	Free text	Criteria used to establish ET diagnosis (e.g., MDS 2018 criteria, Washington Heights-Inwood criteria, clinical diagnosis by movement disorder specialist, not stated).
**Section 2: Technology (Fields 7–10)**			
F7	Device / Platform Name	Free text	Specific device name and model where reported (e.g., 'Wacom Intuos 3 digitising tablet'; 'Kinesia system, Cleveland Medical Devices'; 'Apple Watch Series 4'). Record 'not specified' if unnamed.
F8	Context of Use	Categorical — lab-clinic / home / hybrid	Setting in which data were collected. Supervised home visits by a researcher are classified as lab-clinic, not home. Home requires unsupervised independent use by the patient without researcher presence.
F9	Tasks Performed	Categorical — multi-select: postural hold / kinetic / rest / spiral or handwriting / gait / ADL / other (specify)	All tasks performed during the study. Select all that apply and specify any tasks recorded as 'other'.
F10	Primary	Free text — be	Main quantitative output used as the digital

	Outcome Metric	specific	biomarker. Do not record only 'tremor score' — specify the metric (e.g., 'peak spectral tremor velocity'; 'spiral width variability index'; 'tremor amplitude RMS in mg'; 'AUC of velocity frequency spectrum').
**Section 3: TRACE Tier Evidence (Fields 11–13)**			
F11	TRACE Tier Assigned	Integer 1–5	Highest TRACE tier for which all core criteria are satisfied. Assign the ceiling tier only — do not average or combine criteria across tiers. See Supplementary File 3 for full tier definitions and the Tier 2/3 boundary decision flowchart.
F12	Tier Rationale	Free text — one sentence	Justification for the assigned tier, referencing the specific evidence present in the paper and explaining explicitly why the next tier was not reached (e.g., 'Home accelerometry across 7 days with ICC=0.82 satisfies Tier 3 calendar day separation and ambulatory criteria').
F13	Clinical Anchor Used	Categorical — TETRAS / FTM-TRS / CRST / TRS / Bain-Findley / other validated scale (specify) / clinician estimate (informal) / none	Validated clinical tremor rating scale used as the comparator for digital metric validation. Distinguish formal validated scales from informal clinician estimates — these represent different levels of construct validity evidence.
**Section 4: Construct Validity (Fields 14–15)**			
F14	Scale-anchored Construct Validity	Yes / No + scale name + statistic	Whether the digital metric correlates with a validated clinical rating scale. If Yes, record the scale and correlation statistic (e.g., 'TETRAS r=0.71, p<0.001'). Note whether the anchor was a formal validated scale or an informal clinician estimate — these are distinguished in reporting.
F15	Discrimination Validity	Yes / No + exact group comparisons + statistic	Whether the digital metric distinguishes between diagnostic groups. If Yes, record the precise groups discriminated and the statistic (e.g., 'ET vs. healthy controls AUC=0.91'; 'ET vs. PD sensitivity 84%, specificity 79%'). Do not record Yes alone, the group detail is essential for synthesis.
**Section 5: Responsiveness and Longitudinal Evidence (Fields 16–19)**			
F16	Immediate Responsiveness	Yes / No + intervention type + direction of change	Whether a pre/post change within a single session was demonstrated (e.g., pre/post medication, DBS ON/OFF, ethanol challenge within one visit). If Yes, record the intervention type and direction of change. In-session changes only, not across calendar days.
F17	Longitudinal Data	Yes / No	Whether measurements were taken on at least two separate calendar days with the same participants. In-session repeated

			measurements on the same day do not qualify regardless of the number of time points.
F18	Home / Ambulatory Setting	Yes / No	Whether any data were collected outside a supervised clinical or laboratory visit, with patients using the device independently. Supervised home visits by a researcher are coded No.
F19	Longitudinal Responsiveness	Yes / No + metric and time interval	Whether the digital metric demonstrates sensitivity to change across separated time points. Treatment response, disease progression, or natural variation over time. Test-retest reliability or day-to-day ICC data alone does not qualify; a change signal across time is required.
**Section 6: Regulatory and Interpretability Signals (Fields 20–23)**			
F20	Adherence / Data Completeness Reported	Yes / No	Whether wear-time, session completion rate, or data completeness statistics are quantified and reported. This is a Tier 4 signal, studies reporting this should be flagged accordingly in F24.
F21	Multi-site	Yes / No + number of sites	Whether data were collected across more than one clinical or research site. If Yes, record the number of sites. This is a Tier 4 signal.
F22	MID Reported	Yes / No + metric and value	Whether a Minimal Important Difference for any digital metric is explicitly stated or calculated. If Yes, record the metric name and the MID value (e.g., 'Digitising tablet log tremor amplitude MDC=51% of baseline geometric mean').
F23	PRO Correlation	Yes / No + scale name	Whether the digital metric is correlated with a patient-reported outcome measure (e.g., QUEST, TETRAS-ADL, EQ-5D, SF-36). If Yes, record the PRO scale name and the direction of correlation where reported.
**Section 7: Flags, Notes, Modality, and Sample Size (Fields 24–27 + Summary)**			
F24	Extraction Flags	Categorical — multi-select	Apply all flags that apply: ET subgroup / ET comparator / therapeutic context / multi-visit clinic / feasibility-grade Tier 3 (ET n<15) / Tier 4 signal / contains Tier 1 component / none. See Part B for full flag definitions and disambiguation logic.
F25	Notes	Free text	Any additional relevant information: unusual design features, methodological limitations, informal clinical anchors, bench validation components, within-session adaptation effects, or queries for second reviewer. In particular note: (a) if the clinical anchor is informal rather than validated; (b) if a pre/post treatment component exists within the session; (c) if the paper contains bench validation alongside clinical data.
F26	Modality-Specific	See modality rules below	First assign the paper to one of the six modality categories, then complete the relevant sub-field.

	Sub-field		Where a study employs multiple modalities, assign to the modality most central to the primary study outcome. Full modality assignment rules and sub-field options are provided in the modality table below.
F27	Sample Size Adequacy Note	Categorical descriptor	Descriptive note only — not an exclusion criterion. Assign one descriptor: n<10 (flag for eligibility review under repeated-measures exception); n=10–19 (feasibility-grade, limits generalisability); n=20–49 (moderate, adequate for single-session construct validity claims); n≥50 (adequate for construct validity and reliability claims).
Sum mary	Narrative Summary Paragraph	Free text structured paragraph	One paragraph synthesising the study's contribution: design and technology used; key finding relevant to ET digital biomarker validation; TRACE tier assignment with rationale for why the next tier was not reached; validity contributions (construct and/or discrimination); and the single most important limitation for scoping review purposes. This output supports narrative synthesis and serves as the primary source for the modality-specific results sections.

Field 26 Modality Sub-fields

Once the primary modality category has been assigned, complete the relevant sub-field from the table below. Where a study employs multiple modalities, assign to the modality most central to the primary outcome reported.

**Modality Category**	**F26 Sub-field Label**	**Response Options**
Wearable IMUs & Smartwatches	Sensor Configuration	Accelerometer only / gyroscope only / accelerometer + gyroscope / full IMU / smartwatch / electromagnetic position tracker / optical position tracker — specify axes or degrees of freedom where reported. Note: electromagnetic and optical position tracking devices are classified here because the clinical application (limb kinematic measurement) is equivalent to IMU-based approaches.
Digitised Handwriting & Drawing	Drawing Platform	Digitising tablet / optoelectronic pen / touchscreen tablet (non-pressure-sensing) / touchscreen tablet (pressure-sensing stylus) / other (specify).
Computer Vision & Contactless	Video Capture Method	Smartphone camera / depth sensor (e.g., Kinect) / optical motion capture system / webcam / other (specify). This category includes any system that does not require an on-body sensor attached to the patient.
Surface EMG	Electrode Configuration	Single muscle / multi-muscle, specify muscles recorded where reported (e.g., 'wrist extensors and flexors bilaterally').
Gait & Whole-Body	Sensor Placement	Ankle / waist / wrist / multiple — specify placements (e.g., 'bilateral ankles and lumbar spine'). Include studies

Wearables		using sensors at two or more body sites primarily for mobility and balance assessment.
Acoustic Voice Analysis	Recording Method	High-definition audio recorder / smartphone microphone / clinical-grade microphone / other (specify). Specify task used: sustained vowel emission / other. This category includes any system using acoustic signal capture of the voice to quantify tremor, without requiring on-body contact sensors.

Part B — Decision Guidance for Interpretive Fields

F4 Study Design — Disambiguation Rules

Study design is the field most prone to misclassification. Apply the following categories in sequence:

- **Single-session:** All data collected on the same calendar day, regardless of the number of tasks, time points, or conditions tested within that day. A within-visit group comparison or pre/post intervention is still single-session. This is the most common design in the ET literature.
- **Test-retest:** Primary purpose is estimating reliability or agreement across measurement occasions separated by calendar days. The main statistical output is an ICC, Bland-Altman plot, CV, or similar. Treatment response or disease progression is not the focus.
- **Multi-visit clinic:** Multiple supervised clinic visits on separate calendar days, but not primarily a reliability study and without any home or ambulatory component.
- **Longitudinal:** Repeated measures over time where the primary aim is tracking change, disease progression, treatment response, or natural variation, rather than simply estimating reliability. A change signal is the primary outcome.
- **Interventional:** Treatment or device study with pre/post measurement as the primary design. May be single-session or multi-visit.
- **Feasibility:** Study explicitly designed to assess the practicability of a device or protocol. Typically small sample, short duration, not powered for efficacy or validity.
- **RCT:** Registered randomised controlled trial with pre-specified digital endpoints.

When in doubt: prefer single-session over cross-sectional for within-session group or condition comparisons. Prefer test-retest over longitudinal when the primary output is a reliability statistic without a change signal. For example, a study where ET patients wear a home sensor on two separate days and day-to-day ICCs are the main outcome is test-retest, not longitudinal.

F11 TRACE Tier — The Tier 2/3 Boundary Decision Flowchart

The Tier 2/3 boundary is the most consequential classification decision in the instrument and the most common source of over-assignment to Tier 3. Apply these three questions sequentially to every study reaching at least Tier 2. Stop as soon as a 'No' is reached.

Question 1: Were measurements taken on at least two separate calendar days with the same participants?

- No → Assign Tier 2, regardless of how many measurement occasions occurred within one day. Stop.
- Yes → Proceed to Question 2.

Question 2: Were any measurements taken in a home or ambulatory setting, outside a supervised clinical visit, with the patient using the device independently?

- No → Assign Tier 2 with the multi-visit clinic flag. Do not elevate to Tier 3. Stop.
- Yes → Proceed to Question 3.

Question 3: Is there evidence of temporal performance across those separated home or ambulatory time points — reliability statistics across days, sensitivity to change, or longitudinal tremor tracking?

- At least one criterion met → Assign Tier 3.
- None met → Assign Tier 2 with the flag 'home deployment, no temporal performance data reported'.

F15 Discrimination Validity — Recording Group Comparisons

Record the specific groups discriminated, not merely Yes or No. The following comparison types are meaningfully distinct:

- **ET versus healthy controls** — establishes detection of pathological tremor
- **ET versus a specific disorder (e.g., PD, dystonic tremor)** — establishes diagnostic specificity
- **ET severity subgroups (e.g., moderate vs. severe)** — establishes within-disease sensitivity
- **ET prognostic subgroups (e.g., DBS responders vs. non-responders)** — establishes predictive validity

Record all group pairs tested. Where discrimination was not statistically significant, record this explicitly (e.g., 'ET vs. healthy controls, no significant difference').

F24 Extraction Flags — Two-Question Test for ET Comparator

Before completing F24, apply two questions to every paper:

Question 1: Is the primary research question about ET, or about another disorder for which ET patients serve as a comparator? If the latter, apply the ET comparator flag.

Question 2: Could this study have been conducted without ET patients and still answered its primary question? If yes, the ET comparator flag applies.

ET subgroup and ET comparator are distinct flags. ET subgroup means ET is a genuine co-primary focus alongside another disorder. ET comparator means ET patients are present mainly to validate discrimination. When uncertain, apply ET comparator, it is the more conservative assignment.

Part C — Extraction Flag Definitions

The following flags are applied in Field 24. They do not alter tier assignment but support subgroup and sensitivity analyses.

Flag	When to Apply	Notes and Disambiguation
ET subgroup	ET is not the sole study population but is a genuine focus of analysis, with ET-specific quantitative results reported.	Distinct from ET comparator. ET subgroup = ET is co-primary alongside another disorder. Both disorders are genuine study foci.
ET comparator	ET patients are present primarily to validate discrimination against another disorder that is the true study focus.	Apply after working through the two-question test in Part B. If the study could have been conducted without ET patients and still answered its primary question, apply this flag. Tier is assigned based on ET-specific evidence only.
Therapeutic context	Device's primary purpose is tremor suppression, but an extractable tremor metric (amplitude, power, frequency) is reported before or after intervention.	Include the paper. The measured tremor signal constitutes a digital biomarker irrespective of the therapeutic intent of the device. Flag distinguishes these papers in any monitoring-only subgroup analysis.
Multi-visit clinic	Data collected across separate calendar days but exclusively in supervised clinical settings with no home or ambulatory component.	Assign Tier 2, not Tier 3. Used to distinguish these papers from single-session Tier 2 studies. Important for sensitivity analyses exploring the Tier 2 evidence base.
Feasibility-grade Tier 3	All Tier 3 criteria met but ET n<15.	Tier 3 is assigned but the small sample size limits the generalisability and statistical precision of the longitudinal and ambulatory evidence. Note in F27 as n=10–19 feasibility-grade.
Tier 4 signal	Multi-site data or adherence reporting is present but the study was not pre-registered and does not have a fully defined measurement context with a specified endpoint.	Retain the assigned tier (Tier 3 or lower) — do not elevate to Tier 4. Flag for sensitivity analysis. These papers represent the strongest candidates for Tier 4 promotion in future work.
Contains Tier 1 component	Paper includes both bench or phantom device validation AND ET clinical patient data.	Assign the highest tier achieved in the patient data component. Note the Tier 1 bench component in F25 as it represents dual-level evidence within a single publication.

Part D — Resolved Boundary Cases

The following cases were resolved during extraction for this review. They are provided as worked examples and as precedents for structurally equivalent scenarios in future reviews. Worked examples of how the corresponding TRACE tier rules were applied are provided in Supplementary File 3, Part C.

**Scenario**	**Decision**	**Rationale and Rule**
Repeated-measures exception to n≥10 threshold Haubenberger 2011 (digitising tablet, ethanol challenge, n=9 ET)(2)	INCLUDED — Tier 2	n=9 ET patients but 54 spiral drawings collected across 6 time points per patient within a pre-registered ethanol challenge paradigm, yielding 54 independent observations with a fully pre-specified design. Tier 2 assigned because all measurement occasions occurred within a single calendar day. Rule: a simple cross-sectional n=9 correlation study would NOT qualify under this exception — the exception requires a substantially greater number of independent observations from a pre-specified design.
Therapeutic device with no extractable biomarker metric Maneski 2011 (TremUNA FES, n=3 ET in mixed cohort)(3)	EXCLUDED	Two independent exclusion reasons: (1) ET n=3 in a mixed PD/ET cohort with ET results not reported separately; (2) paper reported only percentage suppression without absolute tremor amplitude or signal values, providing no extractable digital biomarker metric. Either reason alone is sufficient for exclusion.
Single-session multi-timepoint study. Risk of misclassification as longitudinal Haubneberger 2011 E.g., 6 measurement occasions across 75 minutes within a single clinic session(2)	Tier 2 maximum Field 4 = single-session or interventional	Repeated measurements within the same calendar day episode never qualify for Tier 3, regardless of the number of time points or tasks. Rule: calendar day separation is the first and non-negotiable criterion for Tier 3, it must be evaluated before the ambulatory criterion.
Multi-visit clinic study without home component Test-retest reliability across two separate clinic visits on different calendar days e.g. Elble 2017(4)	Tier 2 + multi-visit clinic flag	Calendar day separation alone is insufficient for Tier 3. The ambulatory or home setting criterion must also be met. Studies with multi-visit clinic data are assigned Tier 2 and flagged as 'multi-visit clinic' to distinguish them from single-session Tier 2 studies in sensitivity analyses.
ET used as comparator group for a PD validation study PD is the primary population; ET patients recruited to demonstrate diagnostic discrimination e.g.	INCLUDED if ET n≥10 and ET-specific data extractable — apply ET comparator flag	Apply the two-question test: (A) Is the primary research question about ET or another disorder? (B) Could the study have been conducted without ET patients and still answered its primary question? If ET is the comparator, include the paper, flag it as ET comparator, and assign the tier based on ET-specific evidence only, not the PD evidence that is the study's primary focus.

Sanderson 2020(5)		
Supervised home visit, researcher present during data collection at patient's home e.g. Louis 2012(6)	Context of Use = lab-clinic (not home)	Home / ambulatory (F18) requires unsupervised independent use by the patient. If a researcher or clinician travels to the patient's home and supervises the session, this is functionally equivalent to a clinic visit. The ecological validity advantage of home monitoring derives from the absence of supervision, not merely the location.

References

1. Tricco AC, Lillie E, Zarin W, O'Brien KK, Colquhoun H, Levac D, et al. PRISMA Extension for Scoping Reviews (PRISMA-ScR): Checklist and Explanation. Ann Intern Med. 2018;169(7):467-73.
2. Haubenberger D, Kalowitz D, Nahab FB, Toro C, Ippolito D, Luckenbaugh DA, et al. Validation of digital spiral analysis as outcome parameter for clinical trials in essential tremor. Mov Disord. 2011;26(11):2073-80.
3. Popović Maneski L, Jorgovanović N, Ilić V, Došen S, Keller T, Popović MB, et al. Electrical stimulation for the suppression of pathological tremor. Med Biol Eng Comput. 2011;49(10):1187-93.
4. Elble RJ, Ellenbogen A. Digitizing Tablet and Fahn-Tolosa-Marín Ratings of Archimedes Spirals have Comparable Minimum Detectable Change in Essential Tremor. Tremor Other Hyperkinet Mov (N Y). 2017;7:481.
5. Sanderson JB, Yu JH, Liu DD, Amaya D, Lauro PM, D'Abreu A, et al. Multi-Dimensional, Short-Timescale Quantification of Parkinson's Disease and Essential Tremor Motor Dysfunction. Front Neurol. 2020;11:886.
6. Louis ED, Gillman A, Boschung S, Hess CW, Yu Q, Pullman SL. High width variability during spiral drawing: further evidence of cerebellar dysfunction in essential tremor. Cerebellum. 2012;11(4):872-9.

Citation

When using or adapting this instrument, please cite: Ledingham D, Macerollo A, Pavese N. Essential Tremor and Digital Biomarkers: A Scoping Review Using the TRACE Framework to Map Readiness for Clinical Trials and Routine Practice. Tremor and Hyperkinetic Disorders. [Year]. Supplementary File 2: TRACE-ET Data Charting Instrument.

The instrument is designed to evolve. Researchers who identify new boundary cases or field refinements during application in other movement disorder contexts are encouraged to document these as an extension of Part D when publishing their own reviews.
